# Predicting perceived visual complexity of abstract patterns using computational measures: The influence of mirror symmetry on complexity perception

**DOI:** 10.1371/journal.pone.0185276

**Published:** 2017-11-03

**Authors:** Andreas Gartus, Helmut Leder

**Affiliations:** Department of Basic Psychological Research and Research Methods, Faculty of Psychology, University of Vienna, Vienna, Austria; Universitat de Valencia, SPAIN

## Abstract

Visual complexity is relevant for many areas ranging from improving usability of technical displays or websites up to understanding aesthetic experiences. Therefore, many attempts have been made to relate objective properties of images to perceived complexity in artworks and other images. It has been argued that visual complexity is a multidimensional construct mainly consisting of two dimensions: A quantitative dimension that increases complexity through number of elements, and a structural dimension representing order negatively related to complexity. The objective of this work is to study human perception of visual complexity utilizing two large independent sets of abstract patterns. A wide range of computational measures of complexity was calculated, further combined using linear models as well as machine learning (random forests), and compared with data from human evaluations. Our results confirm the adequacy of existing two-factor models of perceived visual complexity consisting of a quantitative and a structural factor (in our case mirror symmetry) for both of our stimulus sets. In addition, a non-linear transformation of mirror symmetry giving more influence to small deviations from symmetry greatly increased explained variance. Thus, we again demonstrate the multidimensional nature of human complexity perception and present comprehensive quantitative models of the visual complexity of abstract patterns, which might be useful for future experiments and applications.

## Introduction

Complexity is an important concept. Since the birth of complexity science in the 1980s, it has influenced almost every scientific field [1]. However, complexity is hard to define. To begin with, the term complexity has two different usages: We may speak of complex systems regarding complexity as the *quality* that makes a system complex. On the other hand, we also often think of something being more complex than something else. In that case, complexity is used as a *quantity* [2]. The topic of our study is computational methods to model the quantitative complexity of images. However, defining complexity as a quantity is also far from easy, as there are at least two complications: First, there are many different potential measures of complexity (the simplest being just the number of parts of an object, however, the relationship between these parts obviously also contributes to complexity). Second, the measures also depend on the viewer (and/or context), since what counts as a part can be different for different observers [2,3].

Image complexity is also important for many areas of research and technology. For instance, visual complexity might affect perception of elapsed time [4–7] while on the other hand, presentation duration also changes complexity perception [8,9]. Complexity influences aesthetic evaluation during early perceptual analysis of objects of aesthetic interest [10–16]. Furthermore, it is also relevant for the design of technical displays [17–19] icon design [20], website development [21,22], appearance of architecture [23,24], and for many more areas.

In this study, we focus mainly on the prediction of visual complexity of abstract patterns as a quantity perceived by an average or “naïve” observer. In particular, we will compare different measures (and combinations of measures) of visual complexity and analyze their relation to perceived (and judged) average complexity. In addition, although it is not our main focus, we also analyze whether the predictive value of complexity measures varies between different observers. In other words, we explore the relationship between objective properties of the images and the subjective process of perception. However, we restricted our study to mere visual features, and did not investigate the influence of meaning on complexity perception like e.g., [25] did. Therefore, the semantic purport of the abstract patterns we used as stimuli is not addressed, because material was chosen for which it is negligible or at least very low.

Visual complexity has been extensively studied by psychology of perception and other disciplines for about 100 years [18]. The idea of sensory input being perceived as simple or complex evolved from Gestalt psychology [26,27], whose law of “Prägnanz” was reformulated as the “principle of simplicity” [28]. Visual complexity was soon utilized to explain aesthetic preferences in a formal way [29–31]—although these authors have presented fundamentally different aesthetic formulas: While Birkhoff [29] derived his aesthetic measure as order divided by complexity, Eysenck [30,31] conceptualized the formula as order multiplied by complexity.

The psychobiological approach of Berlyne [10,11] assumed that the arousal potential of a stimulus (which is thought to be associated with complexity but also with novelty) is related to its hedonic value by an inverted U-shaped function. However, the results of studies testing these assumptions are mixed and e.g., [32] concluded that inverted U-shaped curves are more an exception than the rule. Some authors have found the proposed inverted U-curves to a certain extent [24,33–36], while others [37–40] have found a linear relation between complexity and aesthetic evaluation. Recently, researchers [41] specifically analyzed individual differences in the relation of liking and complexity. They report that an across-participant analysis led to the proposed inverted U-curve. However, an analysis on the level of individual participants revealed that the inverted U-shape was actually composed from different individual liking functions. Thus, it is possible that the inverted U-curve might be just an artifact of group-level analysis systematically ignoring individual differences.

As an additional complication, already briefly addressed in the first paragraph, visual complexity cannot be conceptualized as the exclusive property of stimulus images. For instance, it is well known that several factors can bias the perception of visual complexity. In Berlyne’s psychobiological theory [10,11], complexity is seen as a collative variable. Such variables are viewed as the result of comparisons within or between stimuli, but also with prior experience. Therefore, experience with a stimulus can change the judgment of its complexity. Specifically, [42] analyzed icon complexity and found a negative correlation of judgments of familiarity with ratings of complexity. Thus, the more familiar an icon appeared, the less complex it was perceived. Consistent with these results, it was found that unfamiliar stimuli are rated more complex than familiar ones [43]. A recent study [44] was able to predict perceived visual complexity with greater-than-chance accuracy from individual EEG (electroencephalography) recordings while participants viewed artworks. Thus, brain imaging techniques are also valuable tools for the study of human complexity perception. Furthermore, it was suggested that asking participants simultaneously for multiple image constructs can also potentially confound complexity ratings [45].

Moreover, it is also not appropriate to describe visual complexity as a single dimension. Instead, it has to be regarded as a multidimensional construct. As described above, empirical evidence for a preference of stimuli with intermediate complexity as proposed in [10,11] is mixed. In an attempt to resolve these problems, Nadal et al. [46] argued that most studies so far treated visual complexity as a one-dimensional property. However, regarding complexity as a multidimensional construct might explain the divergent results of preceding studies. They concluded that two dimensions of visual complexity (amount and variety of elements, and organization and grouping of the elements) can explain most of the variance. In addition, asymmetry might be added as a third dimension. Similar suggestions have been made by earlier researchers. E.g., [35] proposed a perceptual and a cognitive factor of complexity. Chipman [47,48] distinguished between a quantitative factor (related to amount of elements) increasing complexity, and a structural factor (determined by different forms of structural organization, but mostly by symmetry) decreasing complexity. Ichikawa [8] confirmed these two factors and explained them by a fast and a slow cognitive process. In addition, he argued that individual differences in complexity ratings can be explained by different weights of the quantitative and structural aspects of the perceived patterns.

A major aspect of the structural factor of complexity proposed by Chipman [47] and Ichikawa [8] is symmetry. Symmetry is everywhere in the environment and it can be detected fast and efficiently by human vision [49–59]. It is assumed that the human visual system has a preference for mirror symmetry, which is the most salient symmetry. Interestingly, this preference seems to be not shared by Orangutans [60]. In particular, a vertical symmetry axis is easier to detect than a horizontal or obliged axis, and elements close to a symmetry axis are more important than distant elements (although contours can also be important). In sum, human symmetry detection can be characterized to be both robust and sensitive to perturbation [57,59]. On one hand, partly symmetric patterns can be easily distinguished from random patterns. On the other hand, small deviations from perfect symmetry can also be detected without much effort [58,61]. However, violations are easier to detect near the midline of a pattern [51]. Furthermore, McManus [62] speculated that small deviations from symmetry (“a little asymmetry”) might play a role in aesthetics and the arts. In contrast to this, [63] found that slightly asymmetric abstract patterns were (on average) less liked than symmetric patterns by art novices who rated the patterns at their own pace. An important biological form of bilateral (or mirror) symmetry is the form of the human face (and body). Thus, it has been argued that the human preference for symmetry stems from the relevance of symmetry as a biological function signaling health and good genes [34,64–68]. On the other hand, it has been argued that symmetry preference is a mere by-product of visual processing [69]. However, it may also be the case that in fact both explanations are true [70].

Clearly, symmetry is often preferred and evaluated as aesthetically pleasing. Using abstract patterns as stimuli, Jacobsen and Höfel [37–39] found symmetry to be the best predictor for the aesthetic judgment of beauty, while complexity was the second-best predictor. Furthermore, the association of symmetry with positive affective evaluation can also be found using implicit methods like the Implicit Association Test (IAT) [71–73]. There is also evidence that there is an automatic response to symmetry in the brain [74–76]. However, while some studies conclude that symmetry spontaneously elicits positive affect [77], the evidence for an automatic emotional response to symmetry in the strongest sense is not fully conclusive and it seems that aesthetic evaluation also requires some intention [74,78]. An evolutionary algorithm was used in [79] as a new technology to generate patterns based on eye-movement measurements. The authors demonstrate that symmetric patterns also “evolve” when people are simply asked to look at the patterns they prefer. However, the increase of symmetry was larger when people were explicitly asked to look for symmetric patterns.

It was also known for long, that symmetry can influence the perception of complexity. Already Attneave [80] found that symmetric shapes are perceived as less complex than asymmetric shapes with the same number of sides. This can probably be attributed to the increase of redundancy caused by symmetry [81,82]. Furthermore, preference of symmetric patterns over scrambled images was explained by the reduction of complexity that goes along with a higher number of symmetry axes [83]. Thus, symmetry must be considered as an important aspect of visual complexity [40,56,81,84–86].

Attempts to derive visual complexity from image properties often counted elements like e.g. the “indefinitely extended straight lines which contain all the sides of the polygon” [29] (p. 34), the non-parallel sides of polygons [30], or the “number of elements in a pattern” [38] (p. 763). Extending these rather simple counting methods, algorithmic information theory (AIT) [87,88] and Kolmogorov complexity theory [89] tried to provide a mathematical formalization of visual complexity. Essentially, here the visual complexity of an image is defined as the length of the shortest program that can produce it. This measure can be approximated by the resulting file size when applying compression algorithms like ZIP or JPEG (Joint Photographic Expert Group) to an image [17,19,90,91]. In a unique approach, [4,5] combined measures of image edges and local symmetries with fuzzy entropy functions to compute the visual complexity of representational paintings. However, the researchers differentiated only between three a-priori defined complexity classes and exclusively concentrated on perceived time as a subjective measure of complexity. Several objective complexity measures were compared across domains for music, affective pictures, and representational paintings in [92]. The authors demonstrated that the relations of complexity measures with other variables like pleasantness or arousal, as well as with perceived complexity might to some extent be domain-specific [90] and could also vary with gender of the viewers. Recently, a high correlation of perceived and predicted complexity (Spearman’s rank correlation coefficient *r*_s_ = .832) was achieved by applying machine learning (artificial neural networks) to combine a large number of objective predictors of visual complexity [93]. Furthermore, [94] used particle swarm optimization in a similar way to determine optimal weights of a linear combination of predictors of visual complexity of real-world images, and [95] utilized genetic programming to derive a measure for visual complexity of texture images, also considering non-linear combinations of predictors. An obvious advantage of such computational measures of visual complexity is that they are not biased by factors like familiarity of the observer with the images or individual preferences.

In sum, visual complexity has to be considered to be a multidimensional construct, consisting of at least two dimensions: A quantitative factor positively related to complexity and linked to number of elements or similar measures, and a structural factor negatively related to complexity linked to order and mostly determined by symmetry. In addition, there might be considerable individual differences in complexity perception, which are partly determined by previous experience and interpretation of content. The goal of this study is to predict perceived visual complexity of abstract patterns from combinations of objective (computational) properties of the individual images. Our approach is threefold: First, we investigate the structure and dimensionality of perceived visual complexity using easy interpretable linear models. Second, we construct a prediction model using more flexible machine learning algorithms. However, because large individual differences might exist between participants that are ignored when only averaged ratings are used, we additionally analyze differences in complexity perception and compare prediction models between participants.

## General methods

### Overview

In a first step, linear combinations of image parameters are used to predict visual complexity by applying ordinary linear models. Using this approach, it is possible to construct parsimonious models that are easy to interpret. Subsequently, a machine learning algorithm is applied to the data and the prediction results are compared with the linear models. Machine learning approaches can take better advantage of the information contained in the predictors, but the resulting models are more complicated and less easy to interpret. However, they can at least serve as an approximation of the upper limit of prediction quality that is possible using the available image parameters as predictors.

To improve generalizability of the results, two independent sets of black-and-white abstract patterns were used in this work. The first stimulus set (Stimulus Set 1) contains 912 abstract patterns extended from the stimuli used in Gartus and Leder [63]. The second stimulus set (Stimulus Set 2) consists of the 252 abstract patterns introduced by Jacobsen and Höfel [37–39].

The patterns of these two stimulus sets were independently rated for visual complexity by between 29 and 64 participants (see individual method sections for details). For each pattern, the mean of the ratings was taken as the primary measure of perceived visual complexity. Subsequently, several potential objective predictors of visual complexity were computed from the stimulus images. As a first attempt to predict perceived visual complexity, several linear models containing one, two, or three of the predictors were calculated and compared. Then, machine learning was applied using different sets of predictors and the results were compared with the linear models. Finally, separate linear models were calculated for each participant to address individual differences.

### Procedure

#### Participants

Participants were mostly undergraduate psychology students of the University of Vienna who participated for course credit, and a few volunteering graduate psychology students. All gave written informed consent prior to the study and had normal or corrected-to-normal vision. Participants were informed that they could quit the study at any time without further consequences and that the data will be used for scientific publication. All data were fully anonymized. The study lasted about 20–30 min and we did not observe any inconvenience in our participants. Since the study was not in any way harmful or invasive, and did not induce stress in participants, no ethical approval was requested, which is in accordance with the ethical guidelines of the University of Vienna.

#### Rating procedure

The rating of visual complexity was performed using E-Prime 2.0.8.90 (Psychology Software Tools, Inc., Pittsburgh, PA) and 19-inch monitors. Participants saw the images on a neutral light grey background (“silver”) from a distance of about 70 cm. Previously to the rating of visual complexity, participants were shown all images in advance (self-paced browsing of pages showing 42 to 48 patterns) to provide an overview over the range of complexity and to ensure mild familiarity with the patterns. Following [96] and [97], visual complexity was defined as “the amount of detail or intricacy of line in the picture” (German: “das Ausmaß der Details bzw. die Komplexität der Linenführung”). Participants rated all patterns on 5-point scales ranging from 1 = “very simple” (German: “sehr einfach”) to 5 = “very complex” (German: “sehr komplex”). All ratings were self-paced.

#### Calculation of predictors

Like in [92], as well as e.g. [19,91,93], we calculated a wide range of potential predictors of visual complexity from the stimulus images using MATLAB R2013a (The MathWorks, Inc., Natick, Massachusetts, USA) and some selected image processing functions [98].

First, the ratio of compressed file size to image size in pixel was computed for GIF (Graphics Interchange Format), PNG (Portable Network Graphics), TIFF (Tagged Image File Format; Lempel-Ziv-Welch compression), and JPEG (Joint Photographic Expert Group; 90% quality) file compression. Since file compression can be different for the original and an image rotated by 90 degrees [7], we used the average compression rate of both orientations. Then, four types of edge detection were applied to the images: Phase congruency (PHC; phasecong3.m) [98], Canny edge detection (CAN; MATLAB function “edge”) [99], perimeter edge detection (PER; MATLAB function “bwperim”), and root mean square (RMS) contrast [92,100,101]. From the resulting enhanced edge images, mean (MN), standard deviation (SD), as well as the product of mean and standard deviation (MNSD) [100] were calculated. Furthermore, the four types of edge images were also compressed using GIF file compression and the ratio of compressed file size to image size was calculated like above. These calculations led to the following 20 (single and combined) predictors of visual complexity: GIF, PNG, TIF, JPG, PHCMN, PHCSD, PHCMNSD, CANMN, CANSD, CANMNSD, PERMN, PERSD, PERMNSD, RMSMN, RMSSD, RMSMNSD, PHCGIF, CANGIF, PERGIF, RMSGIF.

In addition, four more parameters as described in [102] were calculated: Assessment of preference for balance (APB) [103], deviation of the center of “mass” (DCM; Euclidian distance from geometric center), mirror symmetry (MS) [104], and homogeneity (HG; relative entropy). In [102], these parameters were used as objective measures of balance and aesthetic preference. However, here we employed them as structural measures to predict perceived visual complexity.

Because mirror symmetry turned out to be an important predictor of visual complexity, its calculation is described here in some more detail than the other parameters (see [102] for a comprehensive description). As described in [104], the symmetry of a pattern is considered to be a measure of similarity of pixels on opposite sides of an axis of reflection. Four symmetry axes were taken into account: Horizontal, vertical, main diagonal, and secondary diagonal. For each axis, corresponding pixels that have the same value on both reflection sides are counted and weighted in a way that pixels close to the axis have more influence. Subsequently, pixel counts are normalized to a value between zero and one. Let *m* and *w* be the height and width of an image. Then, to analyze symmetry with respect to the vertical symmetry axis, *n = w/2* comparisons are needed if *w* is even. If *w* is odd, *n = (w-1)/2* comparisons are needed. Let furthermore *X*_*ij*_ be a binary variable that equals 1 if pixels are matching and 0 otherwise. The symmetry *s* of the vertical axis is then calculated as

$$s=\frac{2}{3mn}\overset{m}{\underset{i=1}{\sum }}\overset{n}{\underset{j=1}{\sum }}X_{ij}\left(1+\frac{j-1}{n-1}\right)$$ (1)

After calculating the symmetry for all four axes, the average of all values is taken and multiplied by 100 (i.e., *MS* = 100 (*MS*1 + *MS*2 + *MS*3 + *MS*4)/4), leading to an overall mirror symmetry between zero and 100. A more detailed description of the measure can be found in [104].

### Data analysis

Data was analyzed in *R version 3*.*2*.*3* [105]. Linear model optimizations and corresponding plots were performed using the *leaps package version 2*.*9* [106]. Random forests were calculated using the *randomForest package version 4*.*6–12* [107] using standard settings. Linear mixed effects models were analyzed using the *lme4 package version 1*.*2–12* [108] and the *lmerTest package version 2*.*0–32* [109].

All linear models predicting perceived visual complexity from one, two, or three of the 24 predictors were calculated and compared using *R*^*2*^ (explained variance). Before calculating the models, all predictors were *z*-standardized (*M* = 0, *SD* = 1) to achieve comparable estimates. From these models, the five best for each number of predictors were investigated further. Our goal was to find parsimonious, easy interpretable models that can explain large amounts of variance. Residual plots and Q-Q plots of residuals were visually inspected to review homogeneity of variance (homoscedasticity) and normal distribution of residuals.

Finally, we also applied random forests [110]. Random forests are a machine learning algorithm that use a large number of decision or regression trees, each constructed from a different subset of predictors and data, to predict the output by majority class (in case of classification) or mean output of all trees (in case of regression as used in this work). First, we were using the same predictors for the random forests as in the linear models. That way, we could compare the linear models with the more powerful machine learning models. In addition, we also calculated random forest models using all predictors. Due to problems with multicollinearity, it was not feasible to include all predictors in the linear models. However, the machine learning models provide an estimation of the upper limit of prediction quality which is achievable by using all available information. While the linear models were simply calculated using the full data sets, we have used 10-fold cross-validation for the machine learning models to control for possible overfitting. In 10-fold cross-validation, the data is first split into 10 parts. Nine parts are used for learning and the 10th part is used to test the prediction quality of the algorithm. This is repeated 10 times using each of the 10 data parts only once for testing. By this procedure, predictions for the whole data set are created, while the algorithm always uses different data for training.

In this work, model comparisons are primarily based on Pearson’s product-moment correlation coefficients (*r*) or the square of it (*R*^*2*^, explained variance). However, for the sake of completeness and comparability with other studies, Spearman’s rank correlation coefficient (*r*_s_), root mean square error (RMSE), and mean absolute error (MAE) are also reported.

## Stimulus Set 1

**Fig 1:**
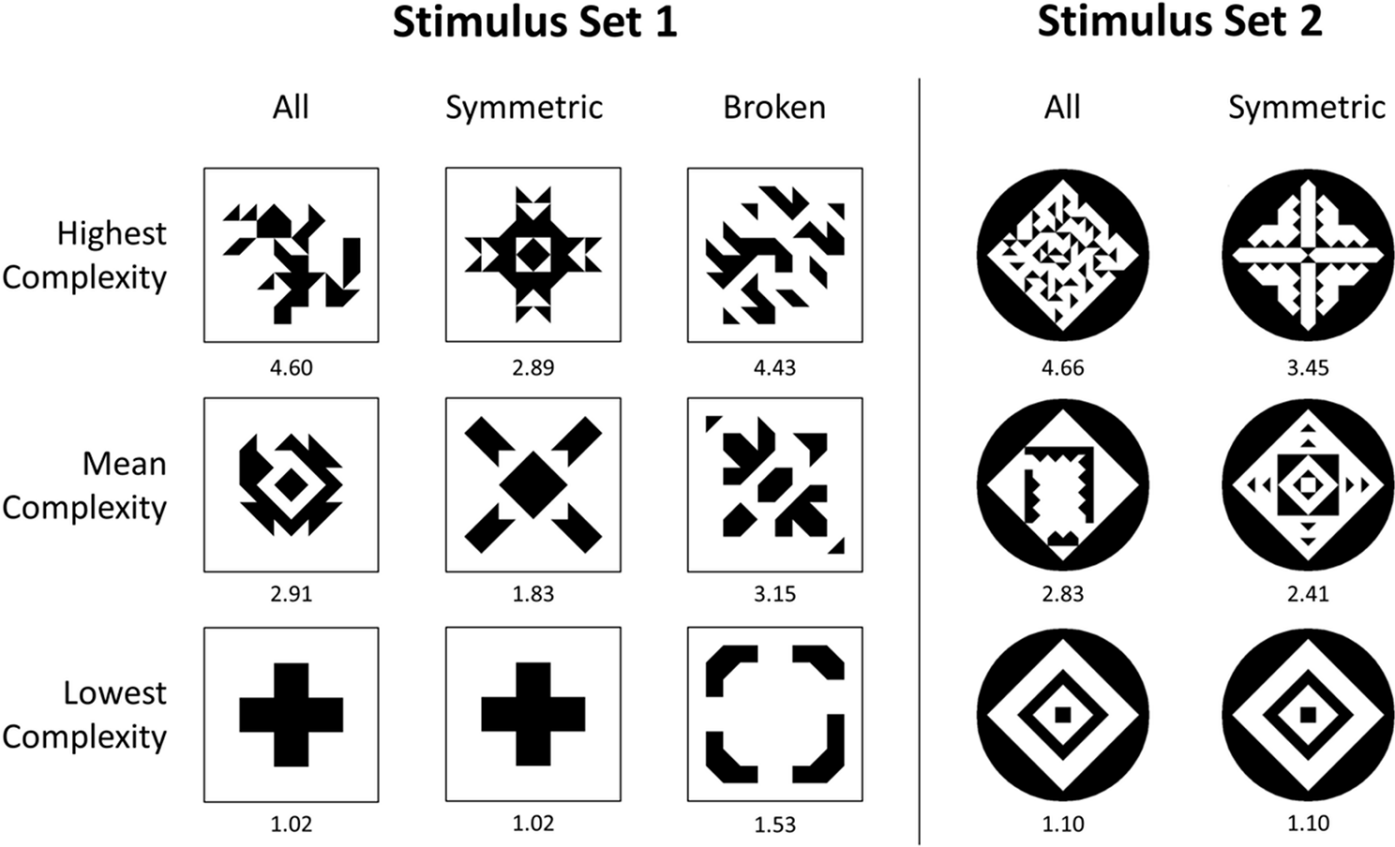
Example patterns from Stimulus Set 1 [63] and Stimulus Set 2 [37–39]. Top row shows the most complex patterns. Middle row shows examples with mean complexity, and bottom row the least complex patterns. Below the patterns, mean complexity ratings are given. For both stimulus sets, three example patterns are depicted for the whole stimulus set (“All”) and exclusively for the symmetric patterns (“Symmetric”). In addition, in case of Stimulus Set 1, example patterns with highest, mean, and lowest complexity are also shown for the group of patterns with broken symmetry (“Broken”). Note that in both stimulus sets, the pattern with the lowest complexity is symmetric, and the pattern with the highest complexity is asymmetric.

The first stimulus set consists of 912 abstract patterns selected from an extended set of patterns that were used by Gartus and Leder [63]. The patterns were generated by a simulated annealing [111] stochastic optimization algorithm programmed in MATLAB. The algorithm placed 36 to 44 black triangular elements in an 8 × 8 rectangular grid on a white background according to several criteria like number of objects and symmetry axes. The 912 selected abstract patterns contain 152 asymmetric and 76 symmetric patterns for each of five types of symmetry (1 or 2 orthogonal, 1 or 2 diagonal, or all 4 symmetry axes). In addition, Stimulus Set 1 also contains a “broken” version of each symmetric pattern. These broken patterns were created from the symmetric patterns by perturbation of a small 3 × 3 area of the pattern, thus producing just-not-symmetric versions of the symmetric patterns. See Fig 1 for example patterns.

### Materials and methods

The 912 abstract patterns were rated in three sessions for visual complexity. The patterns had a size of 600 × 600 pixels corresponding to a physical size of 175 × 175 mm on the monitor. In the first two sessions, two sets of 288 patterns each were rated by 48 (33 women, age range: 20–31 years, *M* = 24.33, *SD* = 2.86) and 64 participants (37 women, age range: 19–45 years, *M* = 25.73, *SD* = 4.57). In the third session, 384 patterns were rated by 47 participants (35 women, age range: 18–29 years, *M* = 21.96, *SD* = 2.51). The third session included 48 patterns already rated in the first two studies. These additional ratings were not used for calculating mean visual complexity.

Initially, we calculated and reviewed the correlation matrix between all predictors together with mean visual complexity. Then, we estimated all linear models using only one, two, or three predictors and compared the models using *R*^*2*^ (explained variance). Finally, we applied machine learning to different sets of predictors and estimated individual linear models for each participant to assess individual differences in complexity perception.

Gartus and Leder [63] showed that small deviations from symmetry do not only lead to a decreased liking, but also considerably increase perceived visual complexity. However, for patterns with broken symmetry, our computational measure of mirror symmetry (MS) is naturally only slightly smaller than the maximum of 100. Thus, a linear combination using this mirror symmetry measure can presumably not account for the rather strong increase of perceived complexity achieved by breaking the symmetry. Therefore, we tried to apply different forms of non-linear transformations to our measure of mirror symmetry. This was done separately for the measures of the four symmetry axes, i.e. before taking the average of the four values and multiplying the result by 100 (compare S1 Fig for some examples of such non-linear transformations.)

### Results and discussion

First, we inspected the correlation matrix of all predictors including mean visual complexity (see S2 Fig for a visualization of this correlation matrix). Table 1 summarizes correlations and corresponding error measures of all predictors with mean visual complexity. Many predictors are highly correlated with each other. In addition, most predictors are positively correlated with mean visual complexity (RMSGIF exhibits the highest correlation with visual complexity of *r* = .634, *p* < .001), and mirror symmetry (MS) is the only predictor which shows a significant negative correlation with visual complexity (*r* = –.578, *p* < .001). In addition, mirror symmetry (MS) and RMSGIF are only slightly negatively correlated with each other (*r* = –.087, *p* = .008). A negative relation of mirror symmetry and visual complexity is also suggested by Fig 1, which shows that the pattern with the lowest complexity is always a symmetric pattern. This is consistent with the well-known fact that symmetric patterns are perceived as less complex than asymmetric patterns [47,48,80,84,86].

**Table 1:** Performance measures of single linear predictors of visual complexity for Stimulus Set 1.

Predictor	*r*	*r*_s_	RMSE	MAE
APB	–.015	.008	0.781	0.641
DCM	.256***	.500***	0.755	0.617
MS	–.578***	–.573***	0.637	0.511
HG	.354***	.307***	0.730	0.605
GIF	.552***	.531***	0.651	0.537
PNG	.315***	.297***	0.741	0.612
TIF	.518***	.490***	0.668	0.553
JPG	.376***	.386***	0.723	0.600
PHCMN	.505***	.476***	0.674	0.556
PHCSD	.469***	.443***	0.689	0.570
PHCMNSD	.485***	.466***	0.683	0.564
CANMN	.514***	.488***	0.670	0.554
CANSD	.521***	.488***	0.666	0.550
CANMNSD	.504***	.488***	0.674	0.558
PERMN	.511***	.454***	0.671	0.551
PERSD	.513***	.454***	0.670	0.550
PERMNSD	.510***	.454***	0.672	0.552
RMSMN	.508***	.477***	0.673	0.554
RMSSD	.509***	.472***	0.672	0.552
RMSMNSD	.504***	.476***	0.675	0.556
PHCGIF	.614***	.590***	0.616	0.507
CANGIF	.552***	.528***	0.651	0.540
PERGIF	.526***	.508***	0.664	0.549
RMSGIF	.634***	.598***	0.604	0.496

*Note*. *r* = Pearson’s product-moment correlation coefficient; *r*_s_ = Spearman’s rank correlation coefficient; RMSE = root mean square error; MAE = mean absolute error. While in case of *r* and *r*_s_ higher is better, for RMSE and MAE lower is better.

* *p* < .05.

** *p* < .01.

*** *p* < .001.

**Fig 2:**
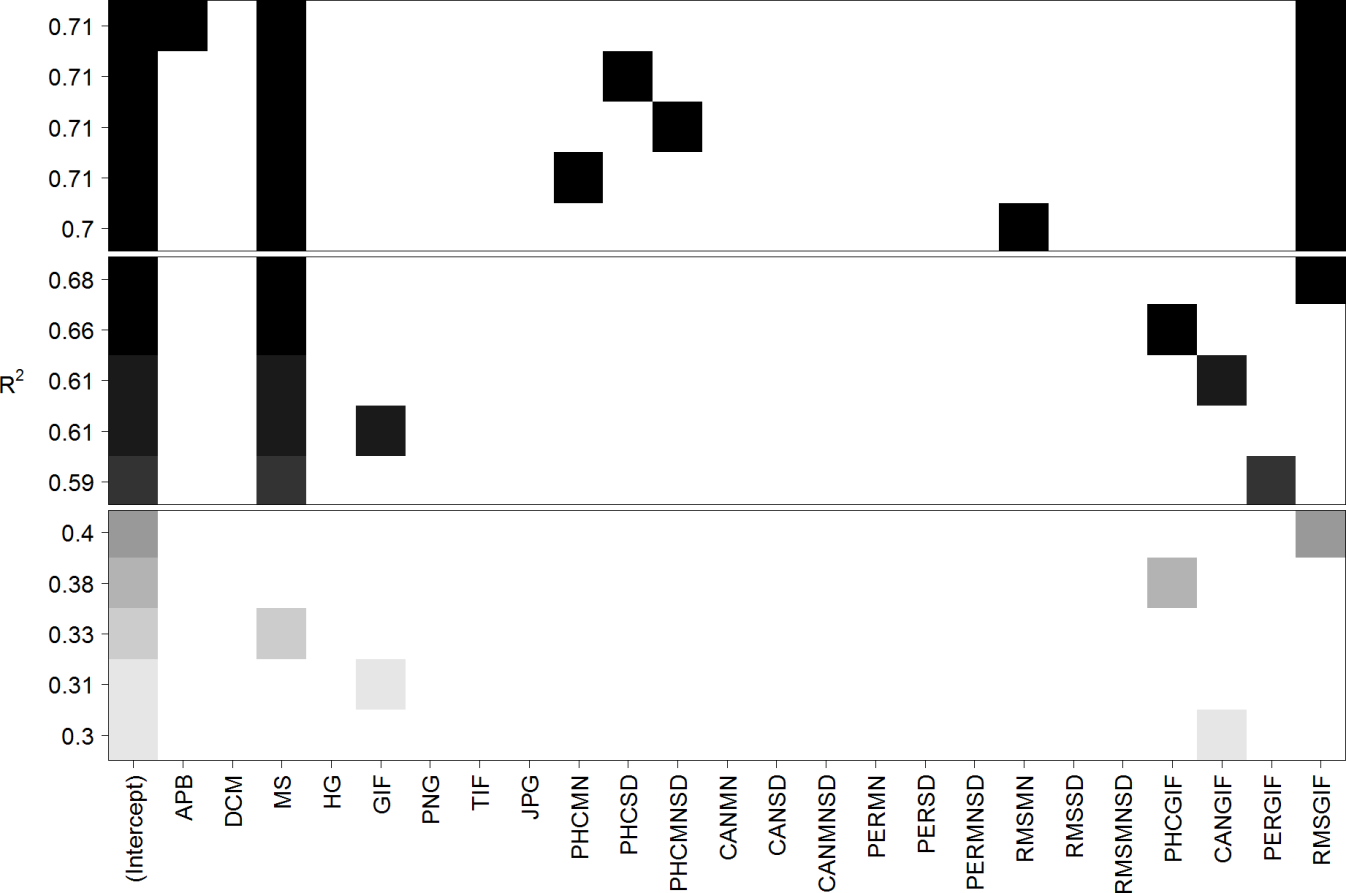
Best five linear models predicting mean visual complexity for Stimulus Set 1 containing one (bottom), two (middle), or three (top) parameters. Each row corresponds to a linear model and squares symbolize the inclusion of a predictor. The quality of the models is evaluated with respect to explained variance (*R*^*2*^). Note that the best single predictor is RMSGIF, the best two-predictor model contains the predictors MS and RMSGIF, and the inclusion of a third predictor only slightly increases the explained variance.

Fig 2 depicts the five best linear models using one, two, or three predictors. As expected from the high correlation, the best single predictor is RMSGIF, which already can explain 40.2% of the variance (see upper part of Table 2 for details). The best model containing two predictors makes use of the predictors mirror symmetry (MS) and RMSGIF and can explain 67.7% of the variance. The inclusion of a third predictor can only slightly increase the explained variance to 71.3%. Thus, the linear model containing mirror symmetry (MS) and RMSGIF can be considered to be the best, most parsimonious and interpretable model (see middle part of Table 2 for details). Inspection of residual plot and Q-Q plot did not show visible inhomogeneity of variance or deviations from normality.

**Table 2:** Linear prediction models of visual complexity for Stimulus Set 1.

	Estimate	*SE*	*t*	*p*
(Intercept)	2.909	0.0200	145.32	< .001***
RMSGIF	0.495	0.0200	24.73	< .001***
*F*(1, 910) = 611.4***, *R*^*2*^ = .4019, adjusted *R*^*2*^ = .4012				
(Intercept)	2.909	0.0147	197.73	< .001***
MS	–0.412	0.0148	–27.85	< .001***
RMSGIF	0.459	0.0148	31.09	< .001***
*F*(2, 909) = 953.8***, *R*^*2*^ = .6773, adjusted *R*^*2*^ = .6766				
(Intercept)	2.909	0.0111	262.02	< .001***
MSA20	–0.506	0.0112	–45.27	< .001***
RMSGIF	0.443	0.0112	39.68	< .001***
*F*(2, 909) = 2018***, *R*^*2*^ = .8162, adjusted *R*^*2*^ = .8158				

*Note*. N = 912. The third linear model contains a non-linear transformation of mirror symmetry (MSA20) instead of the original mirror symmetry (MS) predictor. This largely improves explained variance.

* *p* < .05.

** *p* < .01.

*** *p* < .001.

**Fig 3:**
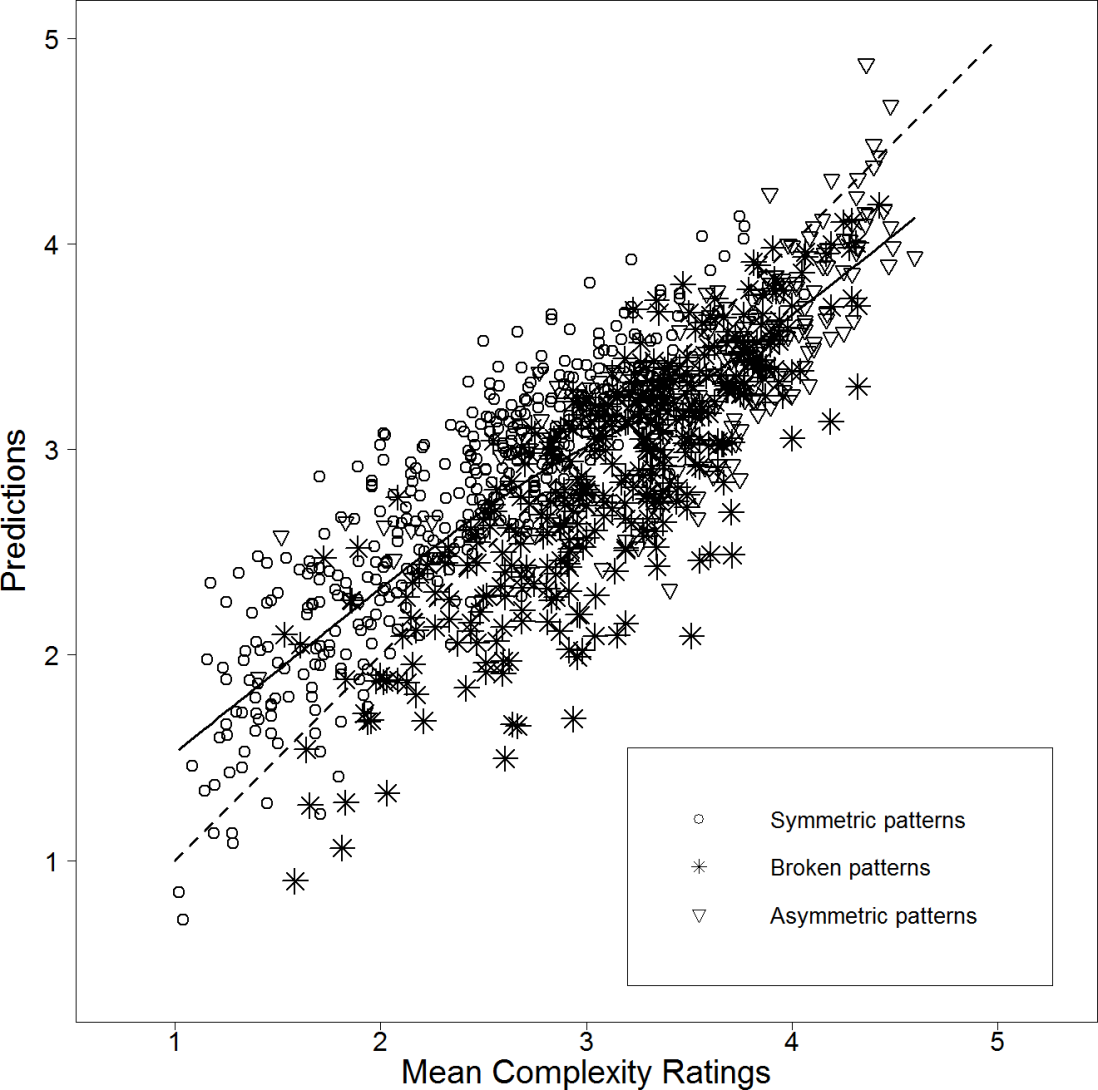
Scatterplot of mean visual complexity versus predictions of the linear model containing mirror symmetry (MS) and RMSGIF as predictors for Stimulus Set 1 (*r* = .823). A LOWESS (locally weighted scatterplot smoothing) [112] regression line—a smooth curve allowing to represent linear and non-linear functions—is used to visualize the linearity of the relation. The diagonal is depicted as dashed line. It can be seen that asymmetric patterns (triangles) are mostly perceived as highly complex. In addition it looks like, that the perceived visual complexity of patterns with broken symmetry (stars) is somewhat underestimated.

Fig 3 shows a scatterplot of mean visual complexity versus the predictions of the two-predictor model. While overall, the scatterplot indicates a quite good linear relation between mean visual complexity and predictions (67.7% of explained variance corresponds to a correlation of *r* = .823), it also suggests that the visual complexity of patterns with broken symmetry might be somewhat underestimated by the linear model.

**Fig 4:**
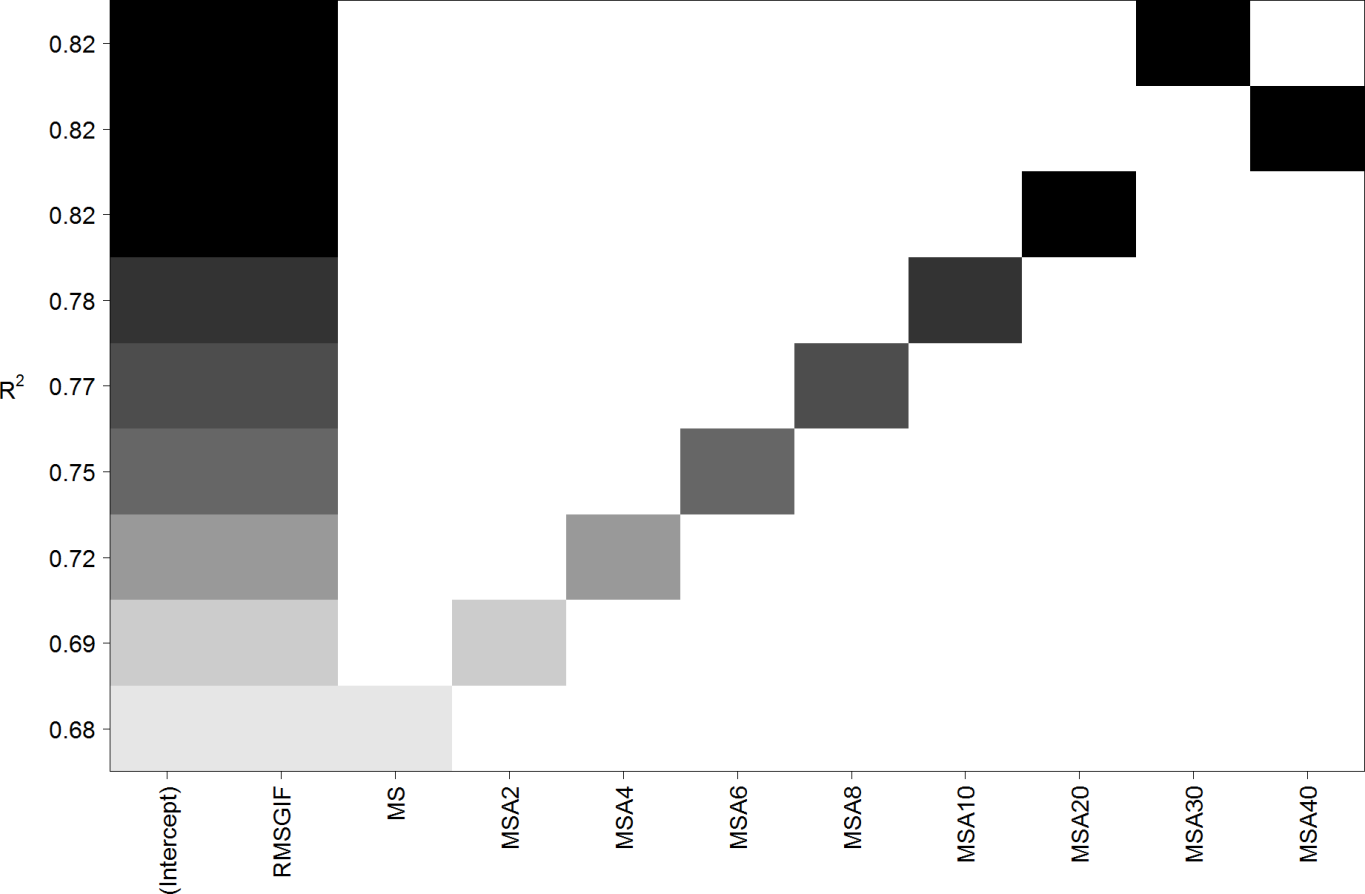
Linear models containing RMSGIF and (transformed and untransformed) measures of mirror symmetry for Stimulus Set 1. Each row corresponds to a linear model and squares symbolize the inclusion of a predictor. The names of the predictors correspond to the power function used to transform them (i.e., MSA6 was transformed by a power function with exponent a = 6; cf. S1 Fig). Note that a power function with exponent a = 20 seems to be a somewhat optimal non-linear transformation. It increases the explained variance (*R*^*2*^) of the model from 68% (MS) to 82% (MSA20).

**Fig 5:**
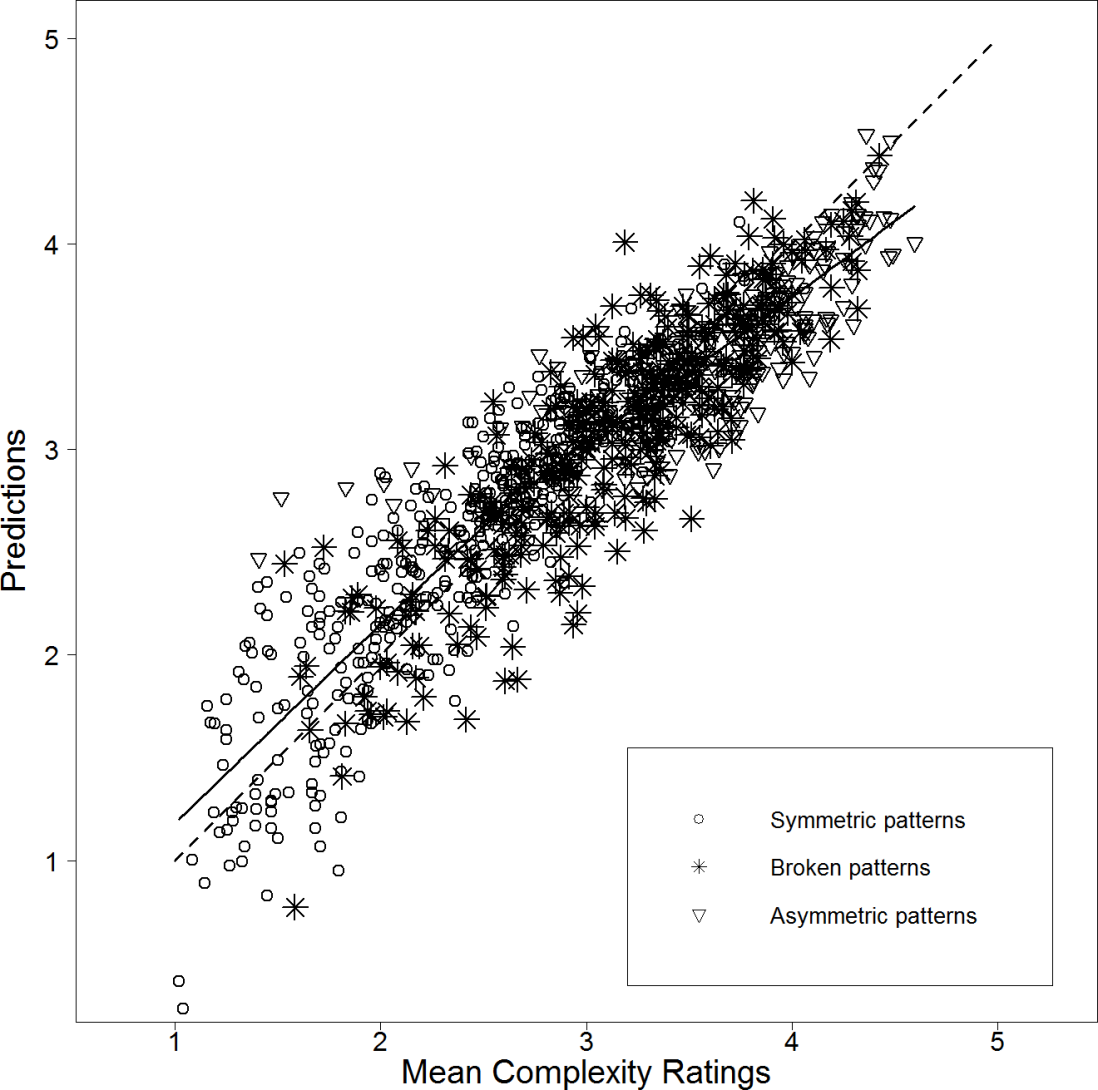
Scatterplot of mean visual complexity versus predictions of the linear model containing mirror symmetry transformed by a power function with exponent a = 20 (MSA20) and RMSGIF as predictors for Stimulus Set 1 (*r* = .903). A LOWESS (locally weighted scatterplot smoothing) [112] regression line—a smooth curve allowing to represent linear and non-linear functions—is used to visualize the linearity of the relation. The diagonal is depicted as dashed line. Note that the perceived visual complexity of patterns with broken symmetry (stars) seems to be no longer underestimated (cf. Fig 3).

Therefore, we applied several non-linear transformations separately to all four axes of our measure of mirror symmetry (MS). Exponential functions led to very similar results as power functions and are thus not discussed any further (cf. S1 Fig). Fig 4 compares (in a similar style as Fig 2) several linear models containing RMSGIF and one of the (transformed or untransformed) measures of mirror symmetry. It can be clearly seen that the non-linear transformation of mirror symmetry increased the explained variance of the model. A somewhat “optimal” transformation could be a power function with an exponent of a = 20 (cf. S1 Fig). Applying this transformation to MS led to the predictor *MSA*20 = 100 (*MS*1^20^ + *MS*2^20^ + *MS*3^20^ + *MS*4^20^)/4, and the explained variance of the model increased from 67.7% to 81.6%. (See also Table 3 and S3 Fig for an overall comparison of all models.) This corresponds to a correlation coefficient of *r* = .903. See lower part of Table 2 for details. The updated scatterplot depicted in Fig 5 reveals that patterns with broken symmetry are no longer visibly underestimated by the new model. However, inspection of residual plot and Q-Q plot did show small signs of inhomogeneity of variance and deviations from normality.

**Table 3:** Comparison of performance measures of prediction models for Stimulus Set 1.

Model	Predictors	*R*^*2*^	*r*	*r*_s_	RMSE	MAE
Linear Model	RMSGIF	.402	.634	.598	0.604	0.496
Linear Model	MS + RMSGIF	.677	.823	.810	0.444	0.363
Linear Model	MSA20 + RMSGIF	.816	.903	.909	0.335	0.266
Linear Model	MS1…4 + RMSGIF	.681	.826	.813	0.441	0.360
Random Forest	MS + RMSGIF	.701	.838	.811	0.427	0.344
Random Forest	MSA20 + RMSGIF	.852	.923	.920	0.300	0.234
Random Forest	MS1. . .4 + RMSGIF	.853	.924	.922	0.314	0.248
Random Forest	MS with ALL	.818	.904	.888	0.335	0.269
Random Forest	MSA20 with ALL	.891	.944	.941	0.261	0.205
Random Forest	MS1. . .4 with ALL	.880	.938	.933	0.277	0.219

*Note*. *R*^*2*^ = *R* squared, *r* = Pearson’s product-moment correlation coefficient; *r*_s_ = Spearman’s rank correlation coefficient; RMSE = root mean square error; MAE = mean absolute error; MSA20 = non-linear transformed predictor of mirror symmetry; MS1…4 = separate predictors for the four axes of mirror symmetry. While in case of *R*^*2*^, *r*, and *r*_s_ higher is better, for RMSE and MAE, lower is better. Note that the different performance measures largely agree.

One has to be careful in calling this power function *the* optimal non-linear transformation, since there exist a huge number of possible non-linear transformations and we obviously could only try a small number of them. Usually, finding the optimal non-linear transformation is much more difficult than in the linear case. It is also worth mentioning that thresholding (which is also a non-linear transformation) the symmetry measures separately for all four axes at a value of 0.95 and then linearly stretching the function above 0.95 to values between zero and one leads to very similar results explaining 81.2% of the variance. In sum, we can state that by applying non-linear transformations that put more emphasis on small deviations from symmetry (and less emphasis on larger differences in the lower range of symmetry), it was possible to considerably increase the explained variance of our linear model predicting perceived visual complexity, which is in line with theory.

Concerning the prediction models using machine learning, one can see in Table 3 (and S3 Fig) that random forest models achieved slightly better, but overall similar results than the linear models using the same predictors. Note that random forest models also profited from the non-linear transformation of mirror symmetry in the same way as linear models. Using MSA20 instead of MS as a predictor increased explained variance from 70.1% to 85.2%. Interestingly, if the random forest was provided with the measures of mirror symmetry separated for the four symmetry axes, it was able to achieve similar prediction quality (85.3% explained variance) than with the already transformed mirror symmetry. Thus, one can assume that in that case, machine learning was able to find the appropriate non-linear transform by itself. (This was expected, since random forests can approximate all kinds of non-linear relationships.) Finally, using all available predictors, random forest models can evidently explain even more of the variance, but the achievement is not very pronounced (maximum explained variance: 89.1%).

To investigate individual differences in perception of visual complexity, we calculated individual linear models for each of the 159 participants. (Note that individual participants did only rate a subset of the patterns in Stimulus Set 1. Thus, differences in the individual linear models might theoretically also be due to different stimulus subsets.) All individual two-predictor models were compared, and those explaining the most variance were chosen (cf. S1 Table, listing all individually best models, their explained variance, and the explained variance of models containing MS and RMSGIF). It can be seen that the explained variance is always smaller than for the averaged data. This is not surprising, because non-averaged ratings are naturally more noisy than mean ratings and can also represent only five discrete steps of complexity (due to the used 5-point rating scale). Thus, they are more difficult to predict. By far the most frequent predictors in the optimized models were mirror symmetry (MS) with 133 and RMSGIF with 120 occurrences. The next frequent predictors were PHCGIF with 34 and APB with 14 occurrences. PHCGIF and RMSGIF are highly correlated (*r* = .961, *p* < .001) and can thus be considered to be very similar predictors. The estimated slopes of MS were in all but two cases (participants 89 and 109) negative and positive in all but one case for RMSGIF (participant 109). In terms of models, 104 of the 159 individually optimized models used MS and RMSGIF as predictors. Additional models using mirror symmetry (MS) and RMSGIF as predictors were also always calculated for each participant and compared with the optimized models. Complexity perception of just a few participants seemed to differ considerably from the average. For instance, a model using APB and PNG as predictors (3.203 + 0.880*APB + 0.337*PNG) could explain 40.0% of the variance in the ratings of participant 77, while the model using MS and RMSGIF could only explain 2.0% of the variance. Participant 109 showed a peculiar optimal model explaining 37.4% of the variance and using MS and RMSGIF, but with differently signed slopes than usually observed (3.462 + 0.527*MS– 0.368*RMSGIF). However, from the optimized models, only nine exhibited more than 5% of additionally explained variance compared to the models using MS and RMSGIF. Thus, for the vast majority of our participants, linear models using MS and RMSGIF as predictors were the best or nearly-best models.

To test for significant individual differences in weights of predictors, we also computed linear mixed effects models (LMMs) using MS and RMSGIF as fixed effects. In addition to random intercepts for stimuli and participants, four models using different random effects structure were calculated and compared (cf. S2 Table, showing the four models and their corresponding AIC and BIC). It turned out that all random slopes were significant (based on likelihood-ratio tests comparing models with and without random slopes). Also, both the Akaike Information Criterion (AIC) and the Bayesian Information Criterion (BIC) clearly indicated that the model using random slopes for both predictors was the best. Therefore, we conclude that there are significant individual differences in the weights of MS and RMSGIF when used to predict perceived visual complexity in Stimulus Set 1.

## Stimulus Set 2

This stimulus set consists of the 252 abstract graphic patterns introduced by Jacobsen and Höfel [37–39]. Some example patterns are presented in Fig 1. It is worth mentioning that Stimulus Set 2 was created independently of Stimulus Set 1 and also differs somewhat in style. However, both stimulus sets are abstract black-and-white patterns placed in a square area (although, in case of Stimulus Set 2, rotated by 45 degrees and put into a black circle; cf. Fig 1). Also, both stimulus sets contain a maximum of four predefined symmetry axes per pattern, and the patterns differ in symmetry and visual complexity. Noteworthy, Stimulus Set 2 is more balanced than Stimulus Set 1, as it contains roughly the same number of symmetric and asymmetric patterns. However, it does contain only few patterns that could be classified as broken symmetric.

### Materials and methods

The 252 abstract patterns from [37–39] were rated by 29 participants (18 women, age range: 18–31 years, *M* = 23.79, *SD* = 3.55) for visual complexity. The patterns had a size of 500 × 500 pixels corresponding to a physical size of 146 × 146 mm on the monitor. Like with Stimulus Set 1, first the correlation matrix of all predictors and mean visual complexity was calculated and inspected. Then, all linear models of one, two, or three predictors were calculated and compared using *R*^*2*^ (explained variance). Different non-linear transformations were applied to the measure of mirror symmetry (separately for all four axes) and the resulting linear models were compared to the best model of step two. Finally, machine learning was applied using different sets of predictors. To further investigate individual differences in complexity perception, all linear models containing two predictors were also calculated and compared separately for each of the 29 participants who rated the patterns of Stimulus Set 2.

### Results and discussion

The correlation matrix of all variables is very similar to Stimulus Set 1 (see Table 4 for performance measures of the individual predictors and S4 Fig for a visualization of the correlation matrix), and thus here not discussed in detail. The predictor showing the maximum correlation with mean visual complexity is again RMSGIF (*r* = .709, *p* < .001) and mirror symmetry (MS) is again negatively correlated with complexity (*r* = –.478, *p* < .001). Mirror symmetry (MS) is also negatively correlated with RMSGIF (*r* = –.127, *p* = .044).

**Table 4:** Performance measures of single linear predictors of visual complexity for Stimulus Set 2.

Predictor	*r*	*r*_s_	RMSE	MAE
APB	–.030	.043	0.829	0.687
DCM	.265***	.391***	0.799	0.666
MS	–.478***	–.495***	0.728	0.603
HG	.227***	.158*	0.807	0.664
GIF	.592***	.591***	0.668	0.538
PNG	.369***	.313***	0.770	0.628
TIF	.569***	.565***	0.682	0.551
JPG	.539***	.527***	0.698	0.564
PHCMN	.600***	.601***	0.663	0.534
PHCSD	.336***	.256***	0.781	0.629
PHCMNSD	.534***	.509***	0.701	0.556
CANMN	.658***	.691***	0.624	0.506
CANSD	.662***	.691***	0.621	0.504
CANMNSD	.653***	.691***	0.628	0.510
PERMN	.666***	.673***	0.618	0.503
PERSD	.670***	.673***	0.615	0.502
PERMNSD	.661***	.673***	0.622	0.505
RMSMN	.680***	.700***	0.608	0.504
RMSSD	.668***	.686***	0.617	0.516
RMSMNSD	.679***	.698***	0.609	0.504
PHCGIF	.639***	.670***	0.637	0.523
CANGIF	.676***	.708***	0.611	0.499
PERGIF	.687***	.701***	0.602	0.492
RMSGIF	.709***	.734***	0.585	0.483

*Note*. *r* = Pearson’s product-moment correlation coefficient; *r*_s_ = Spearman’s rank correlation coefficient; RMSE = root mean square error; MAE = mean absolute error. While in case of *r* and *r*_s_ higher is better, for RMSE and MAE lower is better.

* *p* < .05.

** *p* < .01.

*** *p* < .001.

**Fig 6:**
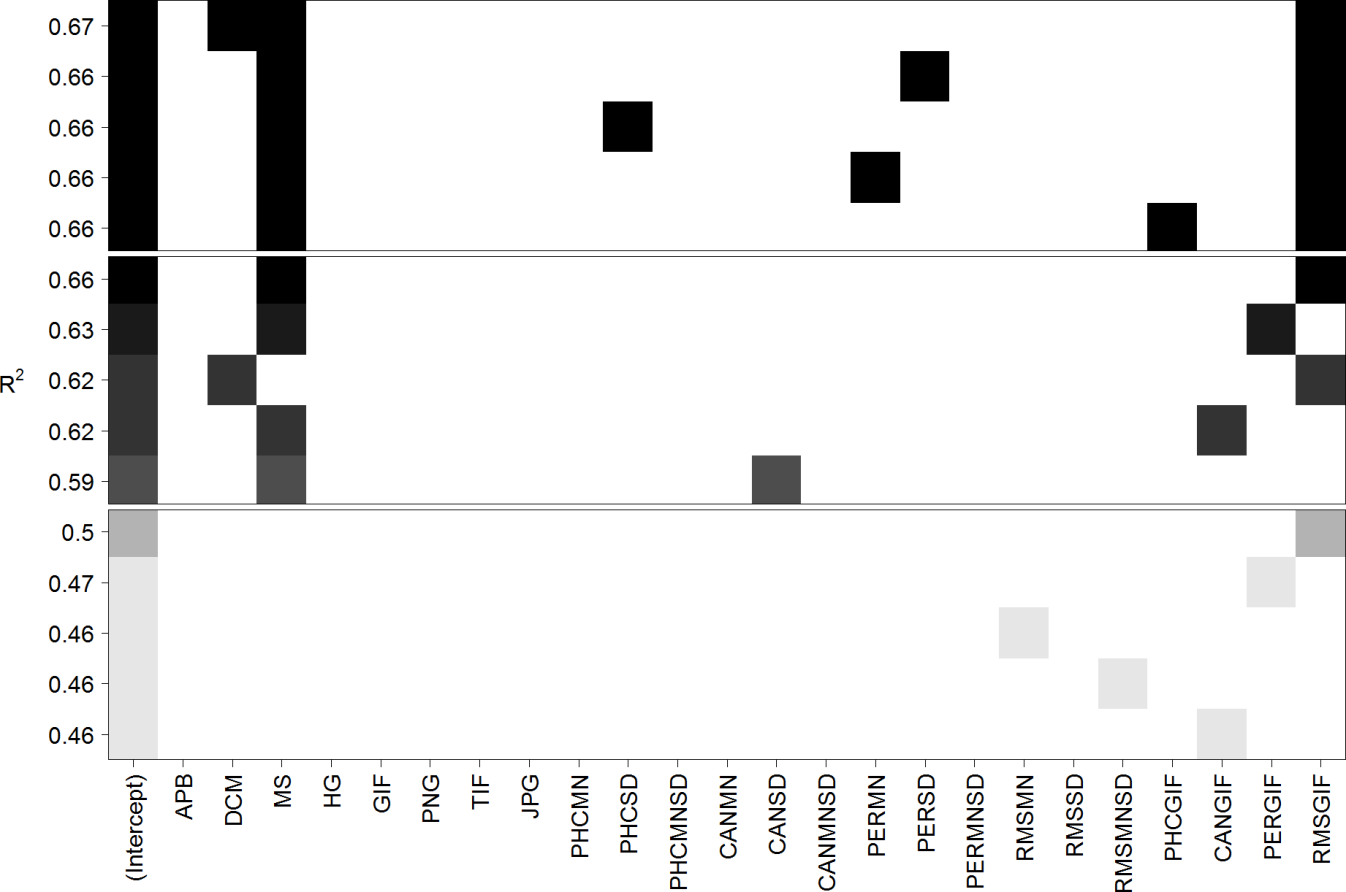
Best five linear models predicting mean visual complexity for Stimulus Set 2 containing one (bottom), two (middle), or three (top) parameters. Each row corresponds to a linear model and squares symbolize the inclusion of a predictor. The quality of the models is evaluated with respect to explained variance (*R*^*2*^). Note that just like for Stimulus Set 1, the best single predictor is RMSGIF, the best two-predictor model contains the predictors MS and RMSGIF, and the inclusion of a third predictor only slightly increases the explained variance.

**Fig 7:**
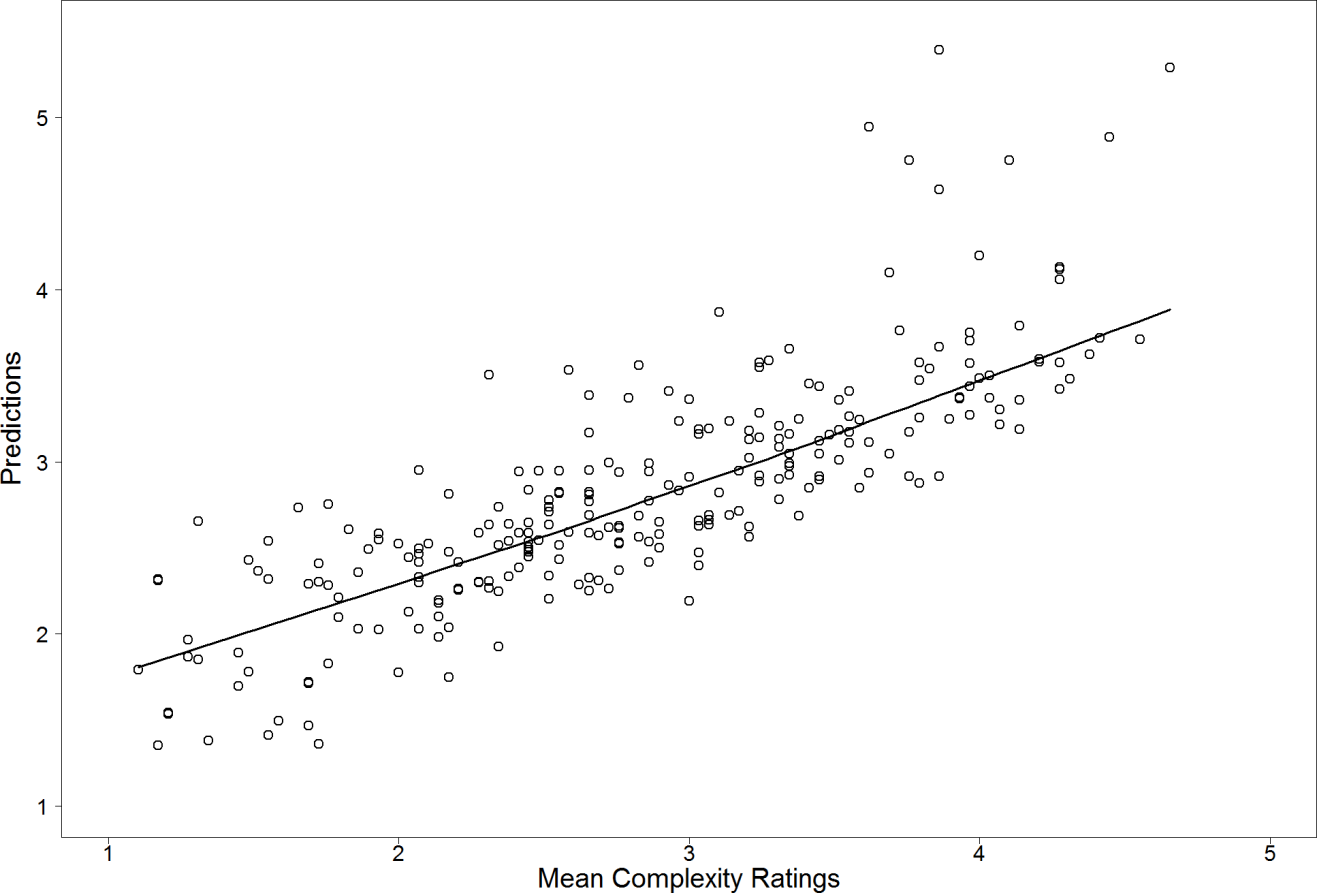
Scatterplot of mean visual complexity versus predictions of the linear model containing mirror symmetry (MS) and RMSGIF as predictors for Stimulus Set 2 (*r* = .810). A LOWESS [112] regression line is used to visualize the linearity of the relation. Note that a few highly complex patterns seem to be overestimated by the linear model.

The five best linear models using on, two, or three predictors were again compared (see Table 5 and Fig 6). Remarkably, the best single predictor is again RMSGIF explaining 50.2% of the variance, and the best model using two predictors is also exactly the same as for Stimulus Set 1 and comprises mirror symmetry (MS) and RMSGIF as predictors (65.6% explained variance). Fig 7 shows a scatterplot of visual complexity versus predictions from the linear model. Inspection of residual plot and Q-Q plot did not show visible inhomogeneity of variance or deviations from normality.

**Table 5:** Linear prediction models of visual complexity for Stimulus Set 2.

	Estimate	*SE*	*t*	*p*
(Intercept)	2.825	0.0370	76.36	< .001***
RMSGIF	0.589	0.0371	15.88	< .001***
*F*(1, 250) = 252.3***, *R*^*2*^ = .5022, adjusted *R*^*2*^ = .5002				
(Intercept)	2.825	0.0308	91.61	< .001***
MS	–0.328	0.0312	–10.53	< .001***
RMSGIF	0.547	0.0312	17.57	< .001***
*F*(2, 249) = 236.9***, *R*^*2*^ = .6555, adjusted *R*^*2*^ = .6528				
(Intercept)	2.825	0.0304	92.98	< .001***
MSA20	–0.336	0.0305	–11.03	< .001***
RMSGIF	0.574	0.0305	18.85	< .001***
*F*(2, 249) = 247.8***, *R*^*2*^ = .6656, adjusted *R*^*2*^ = .6629				

*Note*. N = 252. The third linear model contains a non-linear transformation of mirror symmetry (MSA20) instead of the original mirror symmetry (MS) predictor. This does only minimally improve explained variance.

* *p* < .05.

** *p* < .01.

*** *p* < .001.

**Fig 8:**
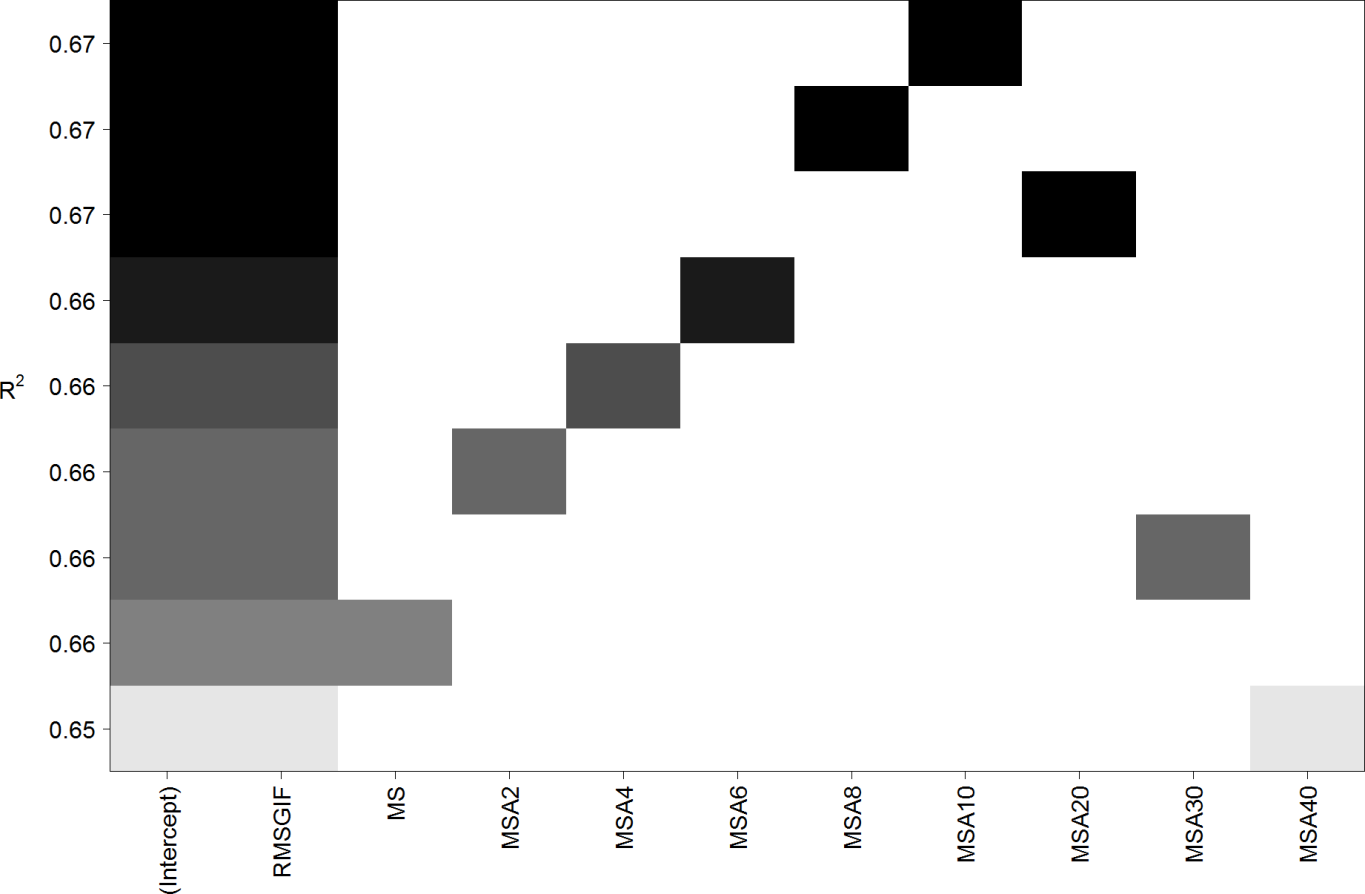
Linear models containing RMSGIF and (transformed and untransformed) measures of mirror symmetry for Stimulus Set 2. Each row corresponds to a linear model and squares symbolize the inclusion of a predictor. The names of the predictors correspond to the power function used to transform them (i.e. MSA6 was transformed by a power function with exponent a = 6; cf. S1 Fig). While similar to Stimulus Set 1, on the first glance there seems to be an optimal non-linear transformation, in contrast to Stimulus Set 1, the explained variance (*R*^*2*^) does practically not improve.

Fig 8 shows the effect of different non-linear transformations of mirror symmetry (MS) on prediction quality of the linear models consisting of RMSGIF and transformed mirror symmetry as predictors. While on the first glance, there seems to be a maximum and an “optimal” transformation similar as for Stimulus Set 1, a second look reveals that the explained variances of the models are practically all the same. Table 6 (and S5 Fig) compares the same kind of models as used for Stimulus Set 1 with each other. It can be seen that all linear models using two predictors (regardless if and how the symmetry predictor was transformed) can explain a similar amount of variance (between 65.6% and 67.2%). Using the non-linear transformed measure of mirror symmetry (MSA20 instead of MS) did only lead to a marginal improvement (66.6% instead of 65.6% explained variance). Inspection of residual plot and Q-Q plot did not show visible inhomogeneity of variance or deviations from normality.

**Table 6:** Comparison of performance measures of prediction models for Stimulus Set 2.

Model	Predictors	*R*^*2*^	*r*	*r*_s_	RMSE	MAE
Linear Model	RMSGIF	.502	.709	.734	0.585	0.483
Linear Model	MS + RMSGIF	.656	.810	.846	0.487	0.389
Linear Model	MSA20 + RMSGIF	.666	.816	.856	0.479	0.377
Linear Model	MS1…4 + RMSGIF	.672	.820	.848	0.475	0.386
Random Forest	MS + RMSGIF	.673	.821	.819	0.474	0.388
Random Forest	MSA20 + RMSGIF	.698	.835	.838	0.456	0.363
Random Forest	MS1…4 + RMSGIF	.692	.832	.840	0.472	0.383
Random Forest	MS with ALL	.758	.871	.867	0.410	0.333
Random Forest	MSA20 with ALL	.771	.878	.878	0.399	0.317
Random Forest	MS1…4 with ALL	.772	.879	.877	0.398	0.318

*Note*. *R*^*2*^ = *R* squared, *r* = Pearson’s product-moment correlation coefficient; *r*_s_ = Spearman’s rank correlation coefficient; RMSE = root mean square error; MAE = mean absolute error; MSA20 = non-linear transformed predictor of mirror symmetry; MS1…4 = separate predictors for the four axes of mirror symmetry. While in case of *R*^*2*^, *r*, and *r*_s_ higher is better, for RMSE and MAE, lower is better. Note that the different performance measures mostly agree.

The marginal improvements achieved by transforming the mirror symmetry predictor can probably be simply explained by the fact that—in contrast to Stimulus Set 1—Stimulus Set 2 contains only very few stimuli that deviate from full symmetry by only a small amount. On the contrary, for each symmetric stimulus, Stimulus Set 1 contains also a corresponding stimulus with broken symmetry. Note however, that non-linear transformation of mirror symmetry did not lead to a decline of explained variance. Thus, one can say that for Stimulus Set 2, non-linear transforming mirror symmetry did not greatly improve predictions, but it also did not worsen them.

Table 6 (and S5 Fig) shows that there are no big differences in explained variance for the linear models and the random forests. All models using just two predictors can explain between 65.6% and 69.8% of the variance. Only the machine learning models using all available predictors can explain a bit more of the variance (between 75.8% and 77.2%). Also, the random forest models show no advantage of transforming the mirror symmetry predictor in a non-linear way. This is most likely due to the same reason as for the linear models: Stimulus Set 2 contains only very few stimuli with broken symmetry.

Like for Stimulus Set 1, we also calculated individually optimized two-predictor linear models for each of the 29 participants (cf. S3 Table, listing all individually best models, their explained variance, and the explained variance of models containing MS and RMSGIF). The explained variance is again always smaller than for the averaged data. The most common individual predictor is RMSGIF, appearing in 20 linear models. The second most common predictor is mirror symmetry (MS), occurring in 19 linear models. Estimated slopes for MS are always negative, while they are positive for RMSGIF. In terms of full models, the most common model with 16 occurrences combines mirror symmetry (MS) and RMSGIF, just like the model for the averaged ratings. (Two other models appear only once and combine MS with predictors highly correlated with RMSGIF: CANGIF (*r* = .924, *p* < .001) and PERGIF (*r* = .929, *p* < .001). Thus, it can be argued that these models are very similar to the ones combining MS and RMSGIF.)

In sum, the most common predictors of the individual linear models are just the same as in the model of the average ratings: RMSGIF and mirror symmetry (MS). Also, the most common model combines these two predictors. Thus, while the major results of the individual analysis confirm the “big picture”, again some individual differences can be found. Roughly one third of the individual models differ from the model of the averaged data. However, it is notable that the majority of the individual linear models still consist of the predictors for mirror symmetry (MS) and RMSGIF (or similar predictors, highly correlated with RMSGIF). Also, the individual linear models explain at the maximum 4.9% more variance than a linear model using MS and RMSGIF as predictors. Therefore, linear models using MS and RMSGIF as predictors were again the best or nearly-best individual models.

As with Stimulus Set 1, we calculated four linear mixed effects models to test for individual difference in weighting the predictors MS and RMSGIF (cf. S4 Table, showing the four models and their corresponding AIC and BIC). Once more, both random slopes were significant and AIC and BIC indicated the model using both random slopes to be the best. Hence, we conclude that significant individual differences in the weighting of MS and RMSGIF also exist for Stimulus Set 2.

## Summary and conclusions

In this study, we sought to predict and explain perceived average visual complexity of abstract patterns and to compare our results with earlier models of visual complexity. Two sets of stimulus images were used (compare Fig 1): A relatively large first set of 912 abstract patterns (Stimulus Set 1) extended from the set originally used by Gartus and Leder [63], also containing broken symmetric patterns. And a smaller second set of 252 abstract patterns (Stimulus Set 2) independently created by Jacobsen and Höfel [37–39], essentially not containing broken symmetric patterns, but being more balanced regarding symmetric vs. asymmetric patterns. The first set was rated for visual complexity by three groups of participants consisting of 48, 64, and 47 people. The second set was only rated by one group of 29 participants. Mean ratings were taken as average perceived complexity of the patterns.

Our first goal was to create parsimonious and interpretable linear models predicting average perceived visual complexity using a wide range of objective properties calculated from the evaluated images. Remarkably, the best linear models for both stimulus sets of abstract patterns consisted of the same two predictors: Mirror symmetry (MS) [102,104] and a combination of root mean square contrast (RMS) [92,100,101] and GIF (Graphics Interchange Format) image compression (RMSGIF). These models could explain 67.7% of the variance in the first (compare Table 3 and S3 Fig), and 65.6% of the variance in the second stimulus set (compare Table 6 and S5 Fig).

Likewise, Chipman [47,48] proposed that perceived visual complexity of a pattern can be explained by two factors: A quantitative variable associated with number of elements and other cumulative aspects setting an upper limit on complexity, and a structural variable representing organization, symmetry, and other similarity transformations present in the patterns, which reduces perceived complexity. Similar results were found by Ichikawa [8], who in addition proposed that individual differences in visual complexity ratings can be characterized by different weights of the structural and the quantitative factor. Moreover, Nadal et al. [46] obtained ratings for 60 stimuli on seven aspects of visual complexity: Unintelligibility of the elements, disorganization, amount of elements, variety of elements, asymmetry, variety of colors, and three-dimensional appearance. A series of discriminant analyses led to three factors of visual complexity: One associated with amount and variety of elements, a second quantifying the way the elements are organized, and finally asymmetry as a third factor. Specifically, color had only little influence on complexity perception, which was also found by [113]. Thus, one could roughly say that they have found one quantitative and two structural factors.

Our results fit well into this picture. For both stimulus sets, the linear models predicting mean perceived visual complexity consist of two predictors: The first predictor is RMSGIF, a variable resulting from a combination of root mean square contrast, enhancing edges and corners of the patterns, with GIF compression rate. This predictor is positively related to complexity. One could consider it to be a quantitative factor. The second predictor is mirror symmetry (MS), which is negatively related to complexity. Thus, it reduces complexity and can be considered to be a structural variable. It should be noted that APB (assessment of preference for balance) and DCM (deviation of the center of mass) can also be characterized as structural factors. However, they seemed to not have played a big role in predicting visual complexity for most of our participants.

Ichikawa [8] proposed in addition, that individual differences in visual complexity ratings can be characterized by different weights of the structural and the quantitative factor. In our results, about two thirds of the best individual linear models explaining perceived visual complexity consisted of two factors (MS and RMSGIF) that can be interpreted like Ichikawa suggested. In the remaining third of the data, the vast majority of models can at most explain 5% more of the variance than the model using MS and RMSGIF as predictors. In addition, linear mixed effects models showed significant random slopes for both predictors with respect to individual participants. Thus, we conclude that explaining individual difference by different weights of a structural and a quantitative factor can be considered a reasonable interpretation. Only for a small minority of our participants, complexity perception must be explained differently. (Another possibility would be of course, that these participants simply did not follow instructions correctly and in essence did not rate patterns for complexity, but for some other property.)

Motivated by the results of [63], which indicated that small deviations from symmetry can have strong effects on aesthetic evaluation and perceived visual complexity, we examined different non-linear transformations of the mirror symmetry (MS) predictor. We applied several power (and exponential) functions (compare S1 Fig) that can be used to transform a linear relation between zero (representing asymmetry) and one (representing full symmetry) to a non-linear relation giving more influence to small deviations from symmetry. It turned out that transforming the mirror symmetry measure (separately for all four symmetry axes) by applying a power function with an exponent of a = 20 could increase the explained variance of the linear model from 67.7% to 81.6% for Stimulus Set 1. However, this effect did practically not exist for Stimulus Set 2, where the explained variance only changed from 65.6% to 66.6% by applying the non-linear transformation to the measure of mirror symmetry. A comparison with models based on machine learning techniques for Stimulus Set 1 reveals that the amount of 81.6% explained variance in the linear model is quite close to the maximum of 89.1% explained variance that could be accomplished by random forest models utilizing all available predictors (compare Table 3 and S3 Fig). For Stimulus Set 2, machine learning models using all available predictors showed a bigger advantage and can explain 77.1% of the variance. But the distance to the linear model explaining 66.6% of the variance is still not particularly high (compare Table 6 and S5 Fig).

These differences between the stimulus sets can probably be explained by the fact that Stimulus Set 1 contains a large number of broken symmetric patterns (380 of 912 patterns) whose perceived visual complexity was presumably underestimated by the linear model using the untransformed version of the mirror symmetry measure (compare Figs 3 and 5). The second stimulus set does however contain only very few patterns that could be described as a broken symmetry. Thus, the predictions were much less affected by the non-linear transformation than in Stimulus Set 1. But it is notable that the predictions at least did not get worse due to the transformation.

One limitation of this study is the fact that our methods were derived from a specific kind of images, namely abstract black-and-white patterns with predefined symmetry axes. However, and in contrast to many other studies, we used two independently created sets of stimuli. And while Stimulus Set 1 might be considered somewhat unbalanced as it contains only a relatively small number of asymmetric patterns, Stimulus Set 2 is more balanced in this respect. Although this, of course, does not completely rule out potential biases in the stimulus sets, it suggests that our results most likely generalize at least to that type of stimuli. In further studies, it would be desirable to extend the approach to a wider class of visual stimuli, like for instance natural textures, architectural images, or abstract artworks. Predictors based on edges, corners, and compression rates can be directly applied to all kinds of images. In addition, for real-world images, prediction accuracy may also benefit from preprocessing steps like removing image noise, standardizing the size of the images, etc. The estimation of mirror symmetry is likely to be more difficult for unrestricted image types, since the predictor used here requires predefined symmetry axes. Yet, general symmetry detection algorithms not depending on such narrow prerequisites are also available (e.g., [114]). Thus, using modified predictors, it might be feasible to extend the methods of this study and e.g. also apply them to abstract artworks or other image types. However, using artworks instead of abstract patterns as stimuli, we would expect less pronounced effects and less accurate predictions, because complexity perception of abstract artworks is presumable also influenced by a large number of individual, not necessary mutually exclusive factors like e.g., art knowledge and interest [12,13,115], familiarity with artworks and style [9,92,116], individual preferences [117–119], cultural differences [60,120], or personality traits [121–126].

In a very first attempt to test our models on more realistic data, we used two texture sets consisting of 54 and 58 images as well as corresponding subjective complexity scores available from [94]. For both textures sets, TIF compression rate was the best single predictor of subjective visual complexity with correlations of *r* = .647 for the first and *r* = .775 for the second set. The mirror symmetry predictor with fixed symmetry axes, modified for grayscale images (MS_GRAY), showed negative correlations with subjective complexity for both texture sets comparable in size to our data sets of abstract patterns (*r* = –.543 and *r* = –.667). However, in stark contrast to our data sets, MS_GRAY and TIF compression rate were also highly negatively correlated in the two texture data sets (*r* = –.701 and *r* = –.793). Therefore, adding MS_GRAY to a linear model already containing TIF as the single predictor could only marginally improve explained variance. In comparison, [94] presented models of complexity perception for both texture sets with a correlation performance of *r* = .81, but did not use symmetry as an explicit predictor. Thus, while mirror symmetry seems to have a similar negative influence on complexity perception in textures as in abstract patterns, the relation between it and quantitative complexity predictors appears to be different. At this point, it is not clear if this is a specific property of the employed data sets, or a more general property of natural and human-made textures. This deserves further investigation using larger, more representative data sets and improved computational predictors.

To summarize, we found that perceived visual complexity of abstract black-and-white patterns with predefined symmetry axes can be parsimoniously predicted by linear models using the following two parameters: 1) A quantitative predictor calculated by a combination of root mean square (RMS) contrast and GIF file compression which is positively related to perceived complexity. Alternatively, some other edge detection methods combined with other file compression algorithms may also work similarly. 2) A structural measure of mirror symmetry negatively related to complexity. Supplementary measures of order and structure like rotational symmetry might play an additional role in other stimulus sets. Non-linear transformation of the symmetry measure in a way that give small deviations from symmetry more weight may improve prediction accuracy by a large amount if stimulus patterns with small deviations from symmetry do exist, but is not supposed to deteriorate prediction accuracy if not. Machine learning algorithms taking more parameters and parameter transformations into account may improve predictions compared to simple linear models, but not by a very large amount. Individual differences in complexity perception do exist, but for the vast majority of participants, linear models using the above mentioned two predictors are sufficient (i.e., almost as good as any other linear model using only two predictors). Our results are in line with the findings of e.g., [8,46,47]. Indeed, we could quantitatively confirm the two-factor structure of perceived visual complexity using a large number of black-and-white abstract patterns from two independently created stimulus sets. It remains a future goal to refine and extend quantitative models of visual complexity to more realistic real-world images and/or artworks, as well as studying corresponding brain activation using non-invasive imaging techniques like fMRI. As mentioned above, this will undoubtedly require taking improved computational measures, but also other additional parameters into account: Meaning of images as well as knowledge and experience of perceivers (obviously not playing a big role for abstract patterns not carrying any semantic meaning) are presumably important factors when judging the complexity of artworks [25]. Thus, most probably, also predictors explaining differences in individual perceivers (and not only in stimulus images) will have to be included in such models.

## Supporting information

S1 Fig Some examples of the non-linear transformation functions applied to the four mirror symmetry measures of the four possible symmetry axes. Considering that x = 0 corresponds to asymmetry, and x = 1.0 to full symmetry, these transforms assign more influence to small deviations from symmetry. Note that power and exponential functions lead to quite similar non-linear transformations.
(TIF)

S2 Fig Correlation matrix of all predictors and mean ratings of visual complexity (“Complexity.MN”) for Stimulus Set 1. Dark and circle-shaped ellipses represent low correlations, while bright and slender ellipses represent high correlations. Right-inclined ellipses depict positive and left-inclined ellipses negative correlations. Note that most predictors correlate positively with mean complexity ratings. One clear exception is mirror symmetry (MS), which exhibits a negative correlation.
(TIF)

S3 Fig Comparison of prediction models for Stimulus Set 1 by explained variance (*R*^*2*^) using data from Table 3. Note that the linear model using non-linear transformed mirror symmetry (MSA20) and RMSGIF as predictors performs almost as good as the random forest models.
(TIF)

S4 Fig Correlation matrix of all predictors and mean ratings of visual complexity (“Complexity.MN”) for Stimulus Set 2. Dark and circle-shaped ellipses represent low correlations, while bright and slender ellipses represent high correlations. Right-inclined ellipses depict positive and left-inclined ellipses negative correlations. Note that the correlations are very similar to the ones in Stimulus Set 1 (cf. S2 Fig).
(TIF)

S5 Fig Comparison of prediction models for Stimulus Set 2 by explained variance (*R*^*2*^) using data from Table 6. Note that in contrast to Stimulus Set 1, the linear model could almost not be improved by using the non-linear transformed mirror symmetry (MSA20) and the random forest models using all predictors perform best regardless of the transformation of the mirror symmetry predictor. This difference might be due to the fact that Stimulus Set 2 does not contain large numbers of broken symmetric patterns.
(TIF)

S1 Table Best two-predictor linear models of visual complexity separate for each participant for Stimulus Set 1. (DOCX)

S2 Table Fixed effects estimates (top), random effects variance estimates (middle), and information criteria (bottom) of linear mixed effects models predicting visual complexity for Stimulus Set 1. (DOCX)

S3 Table Best two-predictor linear models of visual complexity separate for each participant for Stimulus Set 2. (DOCX)

S4 Table Fixed effects estimates (top), random effects variance estimates (middle), and information criteria (bottom) of linear mixed effects models predicting visual complexity for Stimulus Set 2. (DOCX)

S1 Dataset Stimulus properties of Stimulus Set 1. Text file with tab-separated values. First row contains variable names. Variables MSA, MSB, MSC, and MSD are separate mirror symmetry measures of all four axes: MS = 100*(MSA+MSB+MSC+MSD)/4. Some predictors are given in a horizontal (“H…”) and a 90 degrees rotated vertical (“V…”) version. For all calculations, the average of the horizontal and the vertical value was used.
(DAT)

S2 Dataset Participant data and complexity ratings for Stimulus Set 1. Text file with tab-separated values. First row contains variable names.
(DAT)

S3 Dataset Stimulus properties of Stimulus Set 2. Text file with tab-separated values. First row contains variable names. Variables are the same as in S1 Dataset.
(DAT)

S4 Dataset Participant data and complexity ratings for Stimulus Set 2. Text file with tab-separated values. First row contains variable names.
(DAT)
