# Supplementary figures and images for: Predicting perceived visual complexity of abstract patterns using computational measures: The influence of mirror symmetry on complexity perception
Source: PLoS One. 2017 Nov 3;12(11):e0185276. doi: 10.1371/journal.pone.0185276 (PMC5669424; doi:10.1371/journal.pone.0185276)

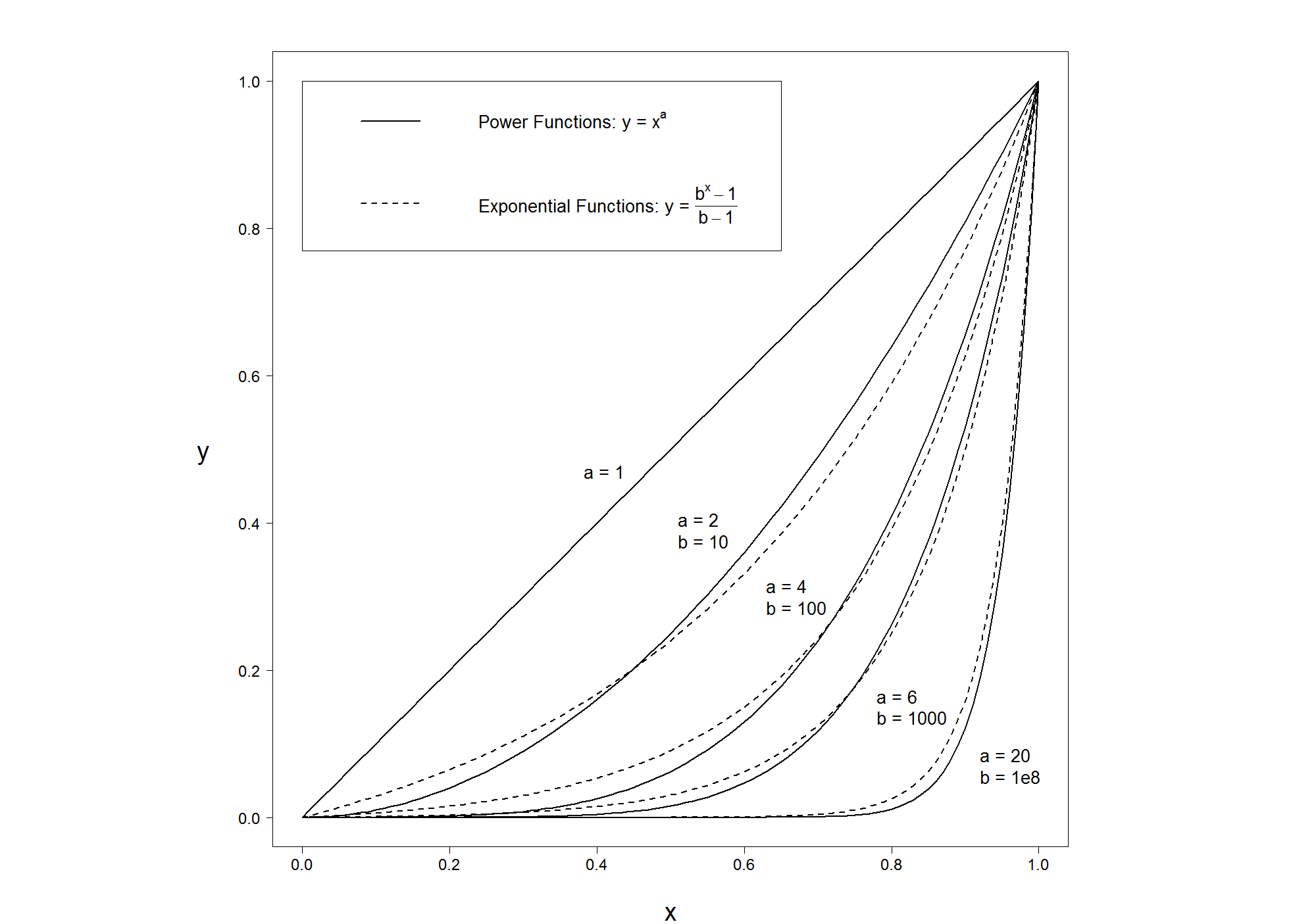

Supplement: S1 Fig — Considering that x = 0 corresponds to asymmetry, and x = 1.0 to full symmetry, these transforms assign more influence to small deviations from symmetry. Note that power and exponential functions lead to quite similar non-linear transformations. (TIF) [file pone.0185276.s001.tif]

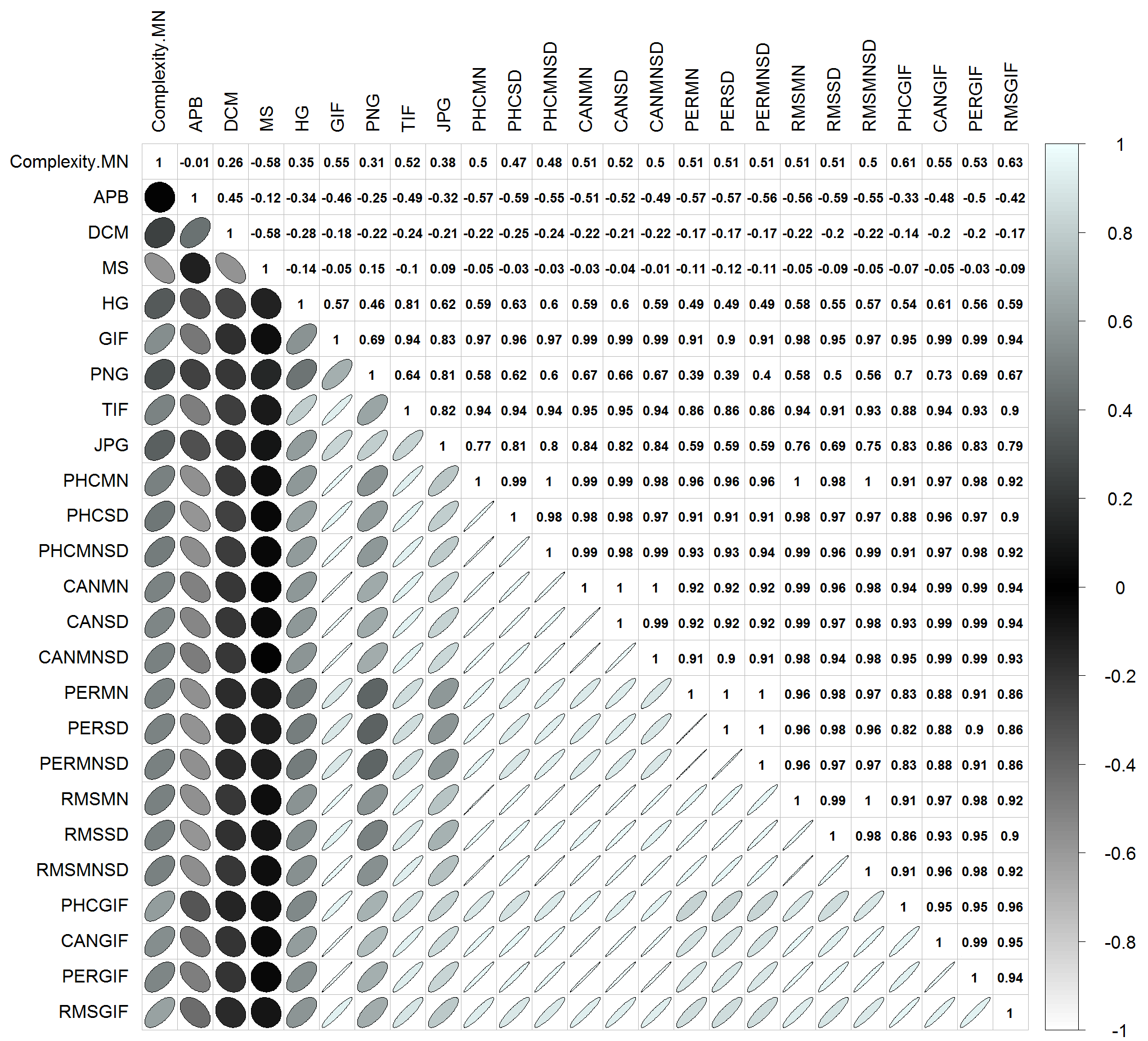

Supplement: S2 Fig — Dark and circle-shaped ellipses represent low correlations, while bright and slender ellipses represent high correlations. Right-inclined ellipses depict positive and left-inclined ellipses negative correlations. Note that most predictors correlate positively with mean complexity ratings. One clear exception is mirror symmetry (MS), which exhibits a negative correlation. (TIF) [file pone.0185276.s002.tif]

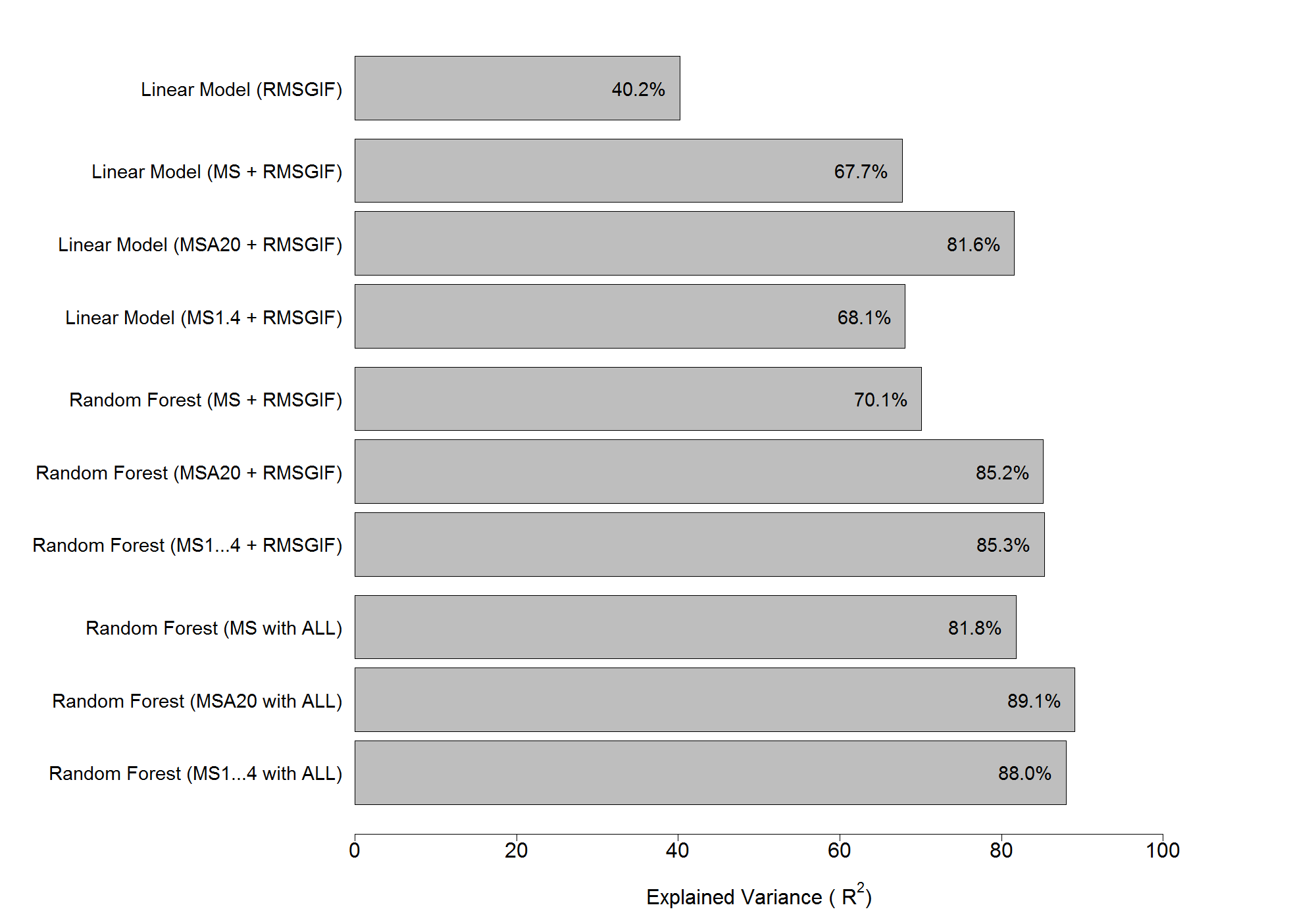

Supplement: S3 Fig — Note that the linear model using non-linear transformed mirror symmetry (MSA20) and RMSGIF as predictors performs almost as good as the random forest models. (TIF) [file pone.0185276.s003.tif]

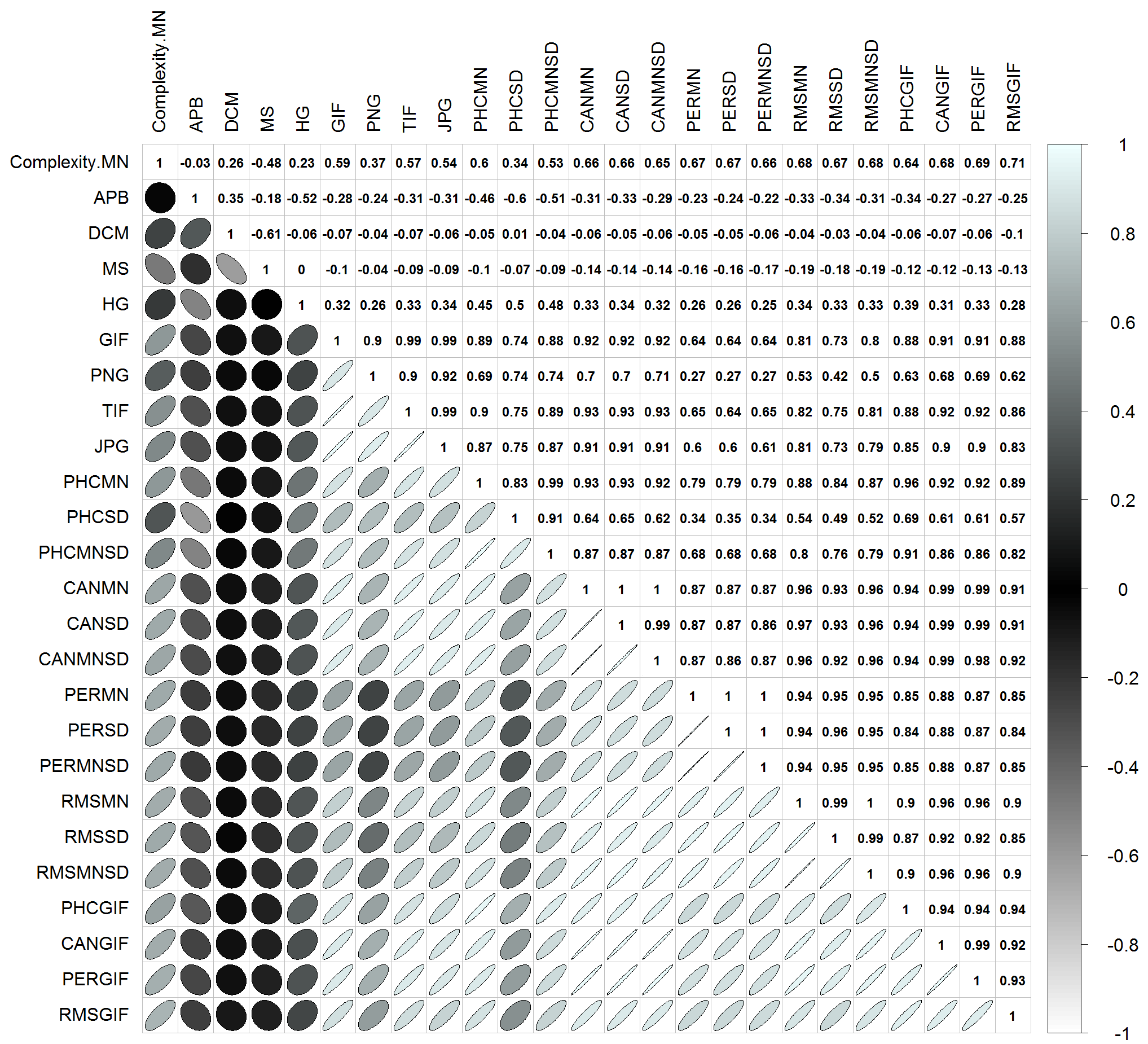

Supplement: S4 Fig — Dark and circle-shaped ellipses represent low correlations, while bright and slender ellipses represent high correlations. Right-inclined ellipses depict positive and left-inclined ellipses negative correlations. Note that the correlations are very similar to the ones in Stimulus Set 1 (cf. S2 Fig). (TIF) [file pone.0185276.s004.tif]

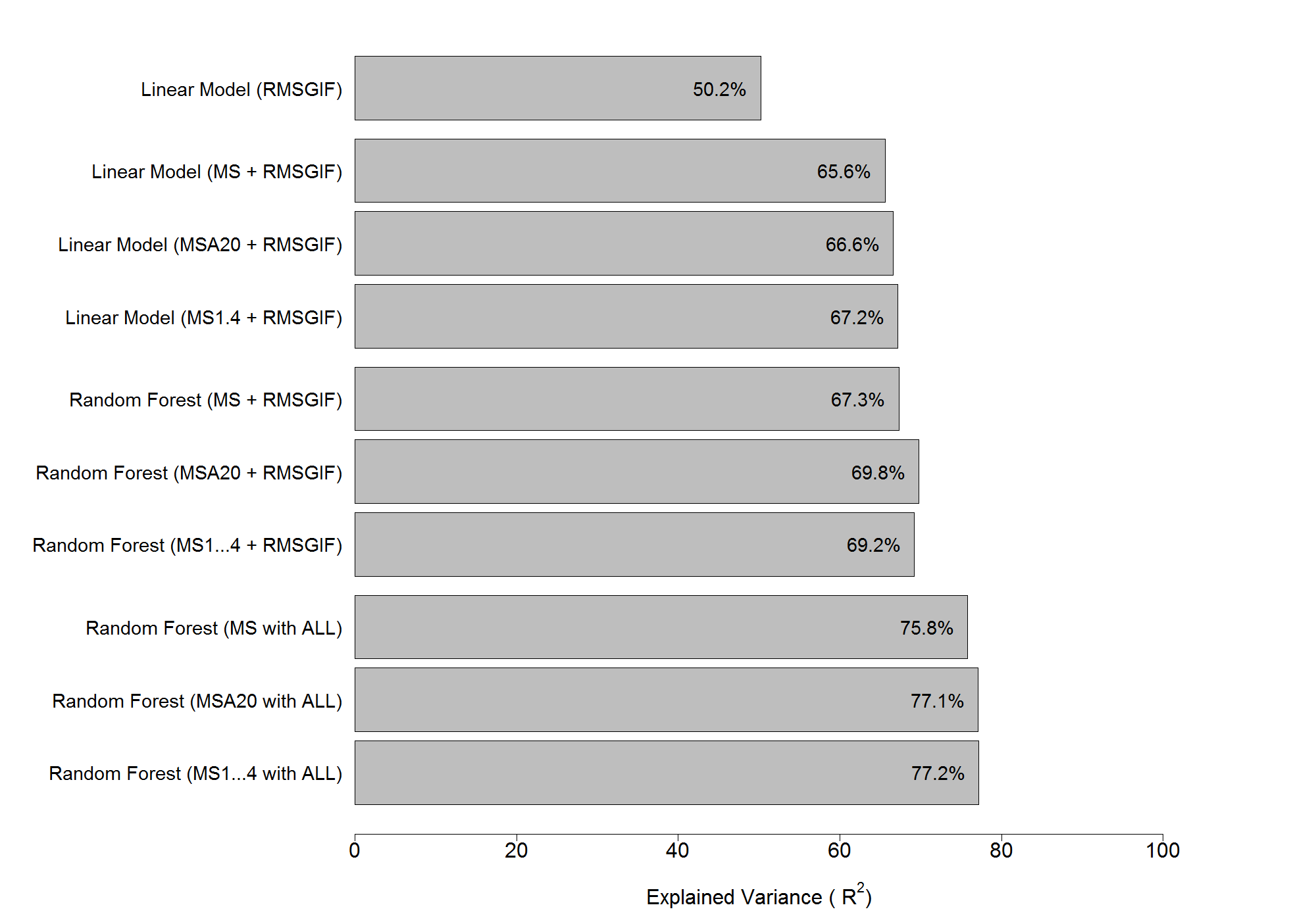

Supplement: S5 Fig — Note that in contrast to Stimulus Set 1, the linear model could almost not be improved by using the non-linear transformed mirror symmetry (MSA20) and the random forest models using all predictors perform best regardless of the transformation of the mirror symmetry predictor. This difference might be due to the fact that Stimulus Set 2 does not contain large numbers of broken symmetric patterns. (TIF) [file pone.0185276.s005.tif]
