# Supplementary material for: Predicting perceived visual complexity of abstract patterns using computational measures: The influence of mirror symmetry on complexity perception
Source: PLoS One. 2017 Nov 3;12(11):e0185276. doi: 10.1371/journal.pone.0185276 (PMC5669424; doi:10.1371/journal.pone.0185276)
Supplement: S1 Table — (DOCX) [file pone.0185276.s006.docx]

**Table S1. Best two-predictor linear models of visual complexity separate for each participant for Stimulus Set 1.**

| **Participant** | **Best two-predictor linear model** | ***R^2^*** | ***R^2^ (MS + RMSGIF)*** |
| --- | --- | --- | --- |
| 1 | 3.135 –0.592*MS +0.503*RMSGIF | 0.5335 | 0.5335 |
| 2 | 2.962 –0.723*MS +0.342*RMSGIF | 0.2673 | 0.2673 |
| 3 | 2.285 +0.283*APB +0.602*PHCGIF | 0.3941 | 0.2666 |
| 4 | 2.927 –0.538*MS +0.432*PHCGIF | 0.2791 | 0.2745 |
| 5 | 3.097 –0.130*MS +0.867*RMSGIF | 0.6738 | 0.6738 |
| 6 | 2.753 +0.389*APB +0.848*PHCGIF | 0.4506 | 0.3711 |
| 7 | 3.309 –0.556*MS +0.356*RMSGIF | 0.4199 | 0.4199 |
| 8 | 3.424 –0.433*MS +0.435*RMSGIF | 0.4753 | 0.4753 |
| 9 | 3.097 –0.685*MS +0.336*RMSGIF | 0.3827 | 0.3827 |
| 10 | 2.896 +0.258*APB +0.611*RMSGIF | 0.4387 | 0.3985 |
| 11 | 3.021 –0.552*MS +0.652*PHCGIF | 0.4997 | 0.4803 |
| 12 | 2.965 –0.494*MS +0.587*RMSGIF | 0.4209 | 0.4209 |
| 13 | 3.295 –0.409*MS +0.724*PHCGIF | 0.5158 | 0.5122 |
| 14 | 3.257 –0.538*MS +0.338*RMSGIF | 0.3464 | 0.3464 |
| 15 | 2.681 –0.446*MS +0.339*RMSGIF | 0.2221 | 0.2221 |
| 16 | 2.531 –0.552*MS +0.538*RMSGIF | 0.3698 | 0.3698 |
| 17 | 2.799 +0.428*APB +0.976*RMSGIF | 0.5252 | 0.4548 |
| 18 | 3.552 –0.267*MS +0.672*RMSGIF | 0.4756 | 0.4756 |
| 19 | 2.313 –0.636*MS +0.302*PHCGIF | 0.2856 | 0.2827 |
| 20 | 3.024 –0.725*CANMNSD +1.107*PHCGIF | 0.3541 | 0.3350 |
| 21 | 2.983 +0.165*DCM +0.611*RMSGIF | 0.5926 | 0.5855 |
| 22 | 2.229 –0.267*MS +0.661*RMSGIF | 0.4832 | 0.4832 |
| 23 | 2.809 –0.691*MS +0.740*RMSGIF | 0.6064 | 0.6064 |
| 24 | 2.604 –0.680*MS +0.132*PHCGIF | 0.2668 | 0.2665 |
| 25 | 3.962 –0.429*MS +0.621*PHCGIF | 0.5219 | 0.5105 |
| 26 | 3.042 –0.474*MS +0.740*RMSGIF | 0.4292 | 0.4292 |
| 27 | 2.139 –0.353*MS +0.252*PHCMNSD | 0.2618 | 0.2594 |
| 28 | 3.056 –0.470*MS +0.339*RMSGIF | 0.4736 | 0.4736 |
| 29 | 2.306 –0.655*MS +0.219*RMSGIF | 0.2500 | 0.2500 |
| 30 | 2.993 +0.139*DCM +0.972*RMSGIF | 0.5889 | 0.5863 |
| 31 | 3.160 –0.347*MS +0.364*RMSGIF | 0.3268 | 0.3268 |
| 32 | 2.691 –0.711*CANMNSD +1.004*PHCGIF | 0.1570 | 0.0905 |
| 33 | 2.785 –0.477*MS +0.298*RMSGIF | 0.3740 | 0.3740 |
| 34 | 2.573 –0.739*MS +0.486*RMSGIF | 0.4202 | 0.4202 |
| 35 | 3.108 –0.626*MS +0.488*RMSGIF | 0.4073 | 0.4073 |
| 36 | 2.066 +0.183*DCM +0.424*RMSGIF | 0.1650 | 0.1609 |
| 37 | 3.705 –0.134*MS +0.294*RMSGIF | 0.3850 | 0.3850 |
| 38 | 2.740 –0.710*MS +0.232*PHCGIF | 0.2621 | 0.2603 |
| 39 | 2.910 –0.454*MS +0.274*RMSGIF | 0.3336 | 0.3336 |
| 40 | 3.007 –0.419*MS +0.303*RMSGIF | 0.2577 | 0.2577 |
| 41 | 2.990 –0.465*MS +0.491*RMSGIF | 0.5140 | 0.5140 |
| 42 | 2.528 –0.442*MS +0.317*PHCGIF | 0.2738 | 0.2694 |
| 43 | 2.587 –0.537*MS +0.383*PHCGIF | 0.4144 | 0.4048 |
| 44 | 2.840 +0.323*APB +0.803*RMSGIF | 0.5135 | 0.5002 |
| 45 | 3.479 –1.299*CANMNSD +1.927*PHCGIF | 0.4922 | 0.4437 |
| 46 | 3.757 –0.519*MS +0.298*PHCGIF | 0.3080 | 0.3060 |
| 47 | 2.934 –0.441*MS +0.459*PHCGIF | 0.4052 | 0.3922 |
| 48 | 2.948 –0.315*MS +0.687*RMSGIF | 0.5482 | 0.5482 |
| 49 | 2.339 +0.208*APB +0.671*RMSGIF | 0.3862 | 0.3722 |
| 50 | 3.563 –0.540*MS +0.549*RMSGIF | 0.4315 | 0.4315 |
| 51 | 3.102 –0.813*PHCMNSD +1.211*PHCGIF | 0.3243 | 0.2670 |
| 52 | 3.354 –0.318*MS +0.480*RMSGIF | 0.3544 | 0.3544 |
| (continued) | | | |
| **Participant** | **Best two-predictor linear model** | ***R^2^*** | ***R^2^ (MS + RMSGIF)*** |
| 53 | 3.003 –0.415*MS +0.518*RMSGIF | 0.4151 | 0.4151 |
| 54 | 2.753 +0.168*APB +0.518*RMSGIF | 0.2275 | 0.2064 |
| 55 | 2.867 –0.315*MS +0.382*RMSGIF | 0.1975 | 0.1975 |
| 56 | 2.682 –0.396*MS +0.219*PHCGIF | 0.1464 | 0.1461 |
| 57 | 2.313 –0.443*MS +0.361*RMSGIF | 0.3368 | 0.3368 |
| 58 | 3.409 –1.438*PHCMNSD +1.895*PHCGIF | 0.3565 | 0.2499 |
| 59 | 2.505 +0.409*APB +0.530*RMSGIF | 0.1335 | 0.0997 |
| 60 | 3.078 –0.635*MS +0.305*RMSGIF | 0.3173 | 0.3173 |
| 61 | 3.451 –0.460*MS +0.559*RMSGIF | 0.3552 | 0.3552 |
| 62 | 2.698 –0.420*MS +0.568*RMSGIF | 0.4303 | 0.4303 |
| 63 | 3.221 –0.652*MS +0.673*RMSGIF | 0.5767 | 0.5767 |
| 64 | 2.539 –0.520*MS +0.548*RMSGIF | 0.5092 | 0.5092 |
| 65 | 3.302 –0.447*MS +0.567*RMSGIF | 0.3341 | 0.3341 |
| 66 | 3.169 –0.406*MS +0.351*PHCGIF | 0.3554 | 0.3448 |
| 67 | 2.615 –0.675*MS +0.559*RMSGIF | 0.4839 | 0.4839 |
| 68 | 2.661 –0.592*MS +0.430*PHCGIF | 0.3281 | 0.3272 |
| 69 | 2.812 –0.448*MS +0.449*RMSGIF | 0.3126 | 0.3126 |
| 70 | 3.039 –0.712*MS +0.543*RMSGIF | 0.4363 | 0.4363 |
| 71 | 2.763 –0.383*MS +0.430*RMSGIF | 0.2761 | 0.2761 |
| 72 | 2.812 –0.415*MS +0.460*RMSGIF | 0.3284 | 0.3284 |
| 73 | 2.987 –0.229*MS +0.514*RMSGIF | 0.4957 | 0.4957 |
| 74 | 2.120 –0.200*MS +0.422*RMSGIF | 0.4309 | 0.4309 |
| 75 | 3.039 –0.528*MS +0.617*RMSGIF | 0.4949 | 0.4949 |
| 76 | 3.570 –0.405*MS +0.494*RMSGIF | 0.3877 | 0.3877 |
| 77 | 3.203 +0.880*APB +0.337*PNG | 0.4003 | 0.0198 |
| 78 | 2.266 –0.410*MS +0.262*RMSGIF | 0.1906 | 0.1906 |
| 79 | 2.661 –0.498*MS +0.611*RMSGIF | 0.5365 | 0.5365 |
| 80 | 3.135 –0.157*MS +0.549*RMSGIF | 0.4109 | 0.4109 |
| 81 | 3.688 –0.574*MS +0.505*RMSGIF | 0.4122 | 0.4122 |
| 82 | 3.232 –0.533*MS +0.623*RMSGIF | 0.4720 | 0.4720 |
| 83 | 3.438 –0.205*MS +0.643*RMSGIF | 0.5413 | 0.5413 |
| 84 | 2.427 –0.545*MS +0.469*RMSGIF | 0.3283 | 0.3283 |
| 85 | 3.508 –0.234*MS +0.288*PHCGIF | 0.2388 | 0.2347 |
| 86 | 3.141 –0.395*MS +0.415*RMSGIF | 0.3657 | 0.3657 |
| 87 | 2.737 –0.304*MS +0.325*RMSGIF | 0.3502 | 0.3502 |
| 88 | 2.898 –0.250*MS +0.498*PHCGIF | 0.3902 | 0.3688 |
| 89 | 3.523 +0.554*MS +0.123*RMSGIF | 0.1895 | 0.1895 |
| 90 | 3.422 –0.622*MS +0.477*RMSGIF | 0.4566 | 0.4566 |
| 91 | 2.201 –0.718*MS +0.318*RMSGIF | 0.2893 | 0.2893 |
| 92 | 2.586 –0.471*MS +0.473*RMSGIF | 0.3785 | 0.3785 |
| 93 | 3.018 –0.296*MS +0.492*PHCMN | 0.4173 | 0.3955 |
| 94 | 2.891 –0.421*MS +0.490*RMSGIF | 0.3726 | 0.3726 |
| 95 | 3.096 –0.552*MS +0.423*RMSGIF | 0.3897 | 0.3897 |
| 96 | 3.431 –0.135*DCM –0.181*JPG | 0.0347 | 0.0106 |
| 97 | 3.198 –0.537*MS +0.684*PHCGIF | 0.4794 | 0.4732 |
| 98 | 3.174 –0.269*MS +0.696*RMSGIF | 0.5686 | 0.5686 |
| 99 | 3.014 –0.611*MS +0.437*RMSGIF | 0.4990 | 0.4990 |
| 100 | 2.771 +0.280*APB +1.037*RMSGIF | 0.5063 | 0.4864 |
| 101 | 3.535 –0.290*MS +0.674*RMSGIF | 0.5614 | 0.5614 |
| 102 | 3.080 –0.364*MS +0.618*RMSGIF | 0.5655 | 0.5655 |
| 103 | 2.854 –0.428*MS +0.372*PHCGIF | 0.4149 | 0.4127 |
| 104 | 2.660 –0.406*MS +0.775*GIF | 0.5523 | 0.5383 |
| 105 | 2.438 –0.656*MS +0.261*RMSGIF | 0.2639 | 0.2639 |
| 106 | 2.618 –0.425*MS +0.463*RMSGIF | 0.4612 | 0.4612 |
| 107 | 2.160 –0.483*MS +0.260*RMSGIF | 0.2260 | 0.2260 |
| 108 | 3.135 –0.542*MS +0.517*RMSGIF | 0.4805 | 0.4805 |
| 109 | 3.462 +0.527*MS –0.368*RMSGIF | 0.3739 | 0.3739 |
| (continued) | | | |
| **Participant** | **Best two-predictor linear model** | ***R^2^*** | ***R^2^ (MS + RMSGIF)*** |
| 110 | 3.080 +0.266*APB +0.804*RMSGIF | 0.5891 | 0.5786 |
| 111 | 2.656 –0.756*MS +0.328*PHCGIF | 0.4289 | 0.4285 |
| 112 | 2.253 +0.306*APB +0.744*RMSGIF | 0.5762 | 0.5425 |
| 113 | 3.170 –0.341*MS +0.380*PHCGIF | 0.3746 | 0.3727 |
| 114 | 2.823 –0.376*MS +0.364*RMSGIF | 0.3593 | 0.3593 |
| 115 | 3.778 +0.344*APB +0.730*RMSGIF | 0.2858 | 0.2614 |
| 116 | 3.108 –0.427*MS +0.354*PHCGIF | 0.4175 | 0.4170 |
| 117 | 3.000 –0.837*PHCSD +0.954*PHCGIF | 0.1942 | 0.0180 |
| 118 | 2.802 –0.590*MS +0.292*RMSGIF | 0.3480 | 0.3480 |
| 119 | 3.000 –0.653*MS +0.439*RMSGIF | 0.5485 | 0.5485 |
| 120 | 2.972 –0.636*MS +0.567*RMSGIF | 0.4501 | 0.4501 |
| 121 | 2.569 +0.260*APB +0.516*RMSGIF | 0.2712 | 0.2152 |
| 122 | 2.788 –0.582*MS +0.488*PHCGIF | 0.3433 | 0.3322 |
| 123 | 3.031 –0.381*MS +0.562*RMSGIF | 0.4910 | 0.4910 |
| 124 | 3.014 –0.346*MS +0.467*RMSGIF | 0.4236 | 0.4236 |
| 125 | 2.583 –0.448*MS +0.612*RMSGIF | 0.3966 | 0.3966 |
| 126 | 2.104 –0.372*MS +0.444*RMSGIF | 0.5353 | 0.5353 |
| 127 | 2.625 –0.439*MS +0.572*RMSGIF | 0.4019 | 0.4019 |
| 128 | 2.625 –0.585*MS +0.335*RMSGIF | 0.3754 | 0.3754 |
| 129 | 2.767 –0.452*MS +0.307*PHCGIF | 0.2086 | 0.2070 |
| 130 | 2.757 –0.571*MS +0.342*RMSGIF | 0.3526 | 0.3526 |
| 131 | 3.597 –0.365*MS +0.608*RMSGIF | 0.4868 | 0.4868 |
| 132 | 2.531 –0.524*MS +0.265*PHCGIF | 0.3294 | 0.3294 |
| 133 | 2.528 –0.437*MS +0.349*RMSGIF | 0.3111 | 0.3111 |
| 134 | 3.090 –0.501*MS +0.718*RMSGIF | 0.6527 | 0.6527 |
| 135 | 2.875 –0.551*MS +0.628*RMSGIF | 0.4150 | 0.4150 |
| 136 | 3.535 –0.603*MS +0.810*RMSGIF | 0.6148 | 0.6148 |
| 137 | 2.872 –0.956*PHCMNSD +1.349*RMSGIF | 0.2470 | 0.2067 |
| 138 | 3.538 –0.707*MS +0.555*RMSGIF | 0.3788 | 0.3788 |
| 139 | 3.014 –0.451*MS +0.632*RMSGIF | 0.4975 | 0.4975 |
| 140 | 1.799 –0.303*MS +0.412*RMSGIF | 0.4716 | 0.4716 |
| 141 | 2.549 –0.426*MS +0.397*RMSGIF | 0.3572 | 0.3572 |
| 142 | 3.042 –0.426*MS +0.494*RMSGIF | 0.4805 | 0.4805 |
| 143 | 3.392 –0.745*MS +0.417*PHCGIF | 0.3524 | 0.3509 |
| 144 | 2.670 –0.389*MS +0.631*RMSGIF | 0.4986 | 0.4986 |
| 145 | 2.212 –0.858*PHCSD +1.196*RMSGIF | 0.3392 | 0.3224 |
| 146 | 1.809 –0.454*MS +0.351*RMSGIF | 0.5007 | 0.5007 |
| 147 | 3.056 –0.433*MS +0.583*RMSGIF | 0.4705 | 0.4705 |
| 148 | 3.014 –0.560*MS +0.312*RMSGIF | 0.3749 | 0.3749 |
| 149 | 2.531 –0.505*MS +0.429*RMSGIF | 0.4911 | 0.4911 |
| 150 | 2.632 –0.549*MS +0.741*RMSGIF | 0.5234 | 0.5234 |
| 151 | 2.556 –0.381*MS +0.647*RMSGIF | 0.6078 | 0.6078 |
| 152 | 2.694 –0.281*MS +0.383*RMSGIF | 0.4445 | 0.4445 |
| 153 | 3.299 –0.444*MS +0.530*RMSGIF | 0.4334 | 0.4334 |
| 154 | 2.899 –0.596*MS +0.558*RMSGIF | 0.4863 | 0.4863 |
| 155 | 1.608 –0.449*MS +0.202*RMSGIF | 0.3712 | 0.3712 |
| 156 | 2.313 –0.488*MS +0.496*RMSGIF | 0.4376 | 0.4376 |
| 157 | 2.740 –0.671*MS +0.388*RMSGIF | 0.4321 | 0.4321 |
| 158 | 2.913 –0.681*MS +0.509*RMSGIF | 0.5241 | 0.5241 |
| 159 | 2.594 –0.573*MS +0.685*PHCGIF | 0.5382 | 0.5345 |

*Note.* *R^2^* = *R* squared. To facilitate comparisons with linear models of the averaged complexity ratings, only two-predictor models were chosen. Note that for all linear models *VIF* < 10, indicating no severe problems with multicollinearity. In addition, the explained variance (*R^2^*) of a linear model containing mirror symmetry (MS) and RMSGIF as predictors is also given.
