# Supplementary material for: Predicting perceived visual complexity of abstract patterns using computational measures: The influence of mirror symmetry on complexity perception
Source: PLoS One. 2017 Nov 3;12(11):e0185276. doi: 10.1371/journal.pone.0185276 (PMC5669424; doi:10.1371/journal.pone.0185276)
Supplement: S2 Table — (DOCX) [file pone.0185276.s007.docx]

**Table S2. Fixed effects estimates (top), random effects variance estimates (middle), and information criteria (bottom) of linear mixed effects models predicting visual complexity for Stimulus Set 1.**

| **Parameter** | **Model 0** | **Model 1** | **Model 2** | **Model 3** |
| --- | --- | --- | --- | --- |
| Fixed effects | | | | |
| Intercept | 2.899*** (0.036) | 2.899*** (0.036) | 2.899*** (0.036) | 2.899*** (0.036) |
| MS | –0.410*** (0.015) | –0.413*** (0.022) | –0.410*** (0.015) | –0.413*** (0.021) |
| RMSGIF | 0.460*** (0.015) | 0.459*** (0.015) | 0.460*** (0.020) | 0.459*** (0.020) |
| Random effects | | | | |
| Stimuli |  |  |  |  |
| Intercept | 0.182 | 0.183 | 0.182 | 0.183 |
| Participants |  |  |  |  |
| Intercept | 0.173 | 0.173 | 0.174 | 0.174 |
| MS |  | 0.040*** |  | 0.038*** |
| RMSGIF |  |  | 0.031*** | 0.030*** |
| Residual | 0.682 | 0.641 | 0.651 | 0.612 |
| AIC | 126661 | 124145 | 124818 | 122267 |
| BIC | 126714 | 124216 | 124888 | 122364 |

*Note.* Standard errors are in parentheses. Number of observations = 50304; Number of stimuli = 912; Number of participants = 159. Significance levels of fixed effects are determined using Satterthwaite’s approximation of degrees of freedom. Significance levels of random effects are calculated using likelihood-ratio tests comparing models with corresponding models not including the random effect. (Since linear mixed effects models cannot be calculated without a random intercept, significance levels of random intercepts are not given.)

* *p* < .05, ** *p* < .01, *** *p* < .001.
