# Supplementary material for: Predicting perceived visual complexity of abstract patterns using computational measures: The influence of mirror symmetry on complexity perception
Source: PLoS One. 2017 Nov 3;12(11):e0185276. doi: 10.1371/journal.pone.0185276 (PMC5669424; doi:10.1371/journal.pone.0185276)
Supplement: S3 Table — (DOCX) [file pone.0185276.s008.docx]

**Table S3. Best two-predictor linear models of visual complexity separate for each participant for Stimulus Set 2.**

| **Participant** | **Best two-predictor linear model** | ***R^2^*** | ***R^2^ (MS + RMSGIF)*** |
| --- | --- | --- | --- |
| 1 | 2.139 –0.620*MS +0.191*RMSGIF | 0.3979 | 0.3979 |
| 2 | 2.960 +0.268*DCM +0.778*RMSGIF | 0.4787 | 0.4742 |
| 3 | 3.270 –0.176*MS +0.826*RMSGIF | 0.5039 | 0.5039 |
| 4 | 2.976 –0.254*PHCMNSD +0.884*RMSGIF | 0.4046 | 0.4030 |
| 5 | 2.552 –0.272*MS +0.413*RMSGIF | 0.3455 | 0.3455 |
| 6 | 3.433 –0.376*MS +0.529*RMSGIF | 0.4203 | 0.4203 |
| 7 | 3.536 –0.382*MS +0.454*RMSGIF | 0.4125 | 0.4125 |
| 8 | 2.726 –0.598*MS +0.772*RMSGIF | 0.4613 | 0.4613 |
| 9 | 3.135 –1.704*CANMNSD +2.526*CANGIF | 0.5329 | 0.5034 |
| 10 | 2.171 –0.405*MS +0.658*RMSGIF | 0.5116 | 0.5116 |
| 11 | 2.976 –0.296*MS +0.206*PERSD | 0.1999 | 0.1766 |
| 12 | 2.718 +0.314*PERSD +0.311*RMSGIF | 0.5002 | 0.4650 |
| 13 | 2.500 –0.578*MS +0.467*CANGIF | 0.4578 | 0.4497 |
| 14 | 3.099 –0.373*MS +0.657*RMSGIF | 0.3588 | 0.3588 |
| 15 | 2.825 –0.439*MS +0.292*RMSGIF | 0.2361 | 0.2361 |
| 16 | 3.171 –0.689*MS +0.419*RMSGIF | 0.4112 | 0.4112 |
| 17 | 2.905 –1.000*CANMNSD +1.655*PERGIF | 0.3775 | 0.3634 |
| 18 | 2.821 –0.801*MS +0.537*PERGIF | 0.3762 | 0.3605 |
| 19 | 2.762 –0.462*MS +0.691*RMSGIF | 0.4202 | 0.4202 |
| 20 | 2.623 +2.584*PHCMN –1.957*PHCMNSD | 0.5701 | 0.5210 |
| 21 | 2.302 –0.755*MS +0.326*RMSGIF | 0.6203 | 0.6203 |
| 22 | 2.274 –0.201*MS +0.518*RMSGIF | 0.3693 | 0.3693 |
| 23 | 2.742 +3.915*PERMN –3.565*PERMNSD | 0.3267 | 0.2835 |
| 24 | 3.056 –0.571*MS +0.369*RMSGIF | 0.4874 | 0.4874 |
| 25 | 3.056 –0.631*MS +0.635*RMSGIF | 0.5545 | 0.5545 |
| 26 | 3.044 –0.383*MS +0.482*RMSGIF | 0.3208 | 0.3208 |
| 27 | 2.504 –1.188*CANMN +1.731*CANGIF | 0.4481 | 0.4350 |
| 28 | 2.440 –1.170*CANMNSD +1.795*CANGIF | 0.4823 | 0.4478 |
| 29 | 3.198 +0.346*PERSD +0.572*RMSGIF | 0.5761 | 0.5536 |

*Note.* *R^2^* = *R* squared. To facilitate comparisons with linear models of the averaged complexity ratings, only two-predictor models were chosen. Note that for the best linear models 9, 17, 20, 23, 27, and 28, *VIF* > 10, indicating problems with multicollinearity. In addition, the explained variance (*R^2^*) of a linear model containing mirror symmetry (MS) and RMSGIF as predictors is also given.
