# Supplementary material for: Predicting perceived visual complexity of abstract patterns using computational measures: The influence of mirror symmetry on complexity perception
Source: PLoS One. 2017 Nov 3;12(11):e0185276. doi: 10.1371/journal.pone.0185276 (PMC5669424; doi:10.1371/journal.pone.0185276)
Supplement: S4 Table — (DOCX) [file pone.0185276.s009.docx]

**Table S4. Fixed effects estimates (top), random effects variance estimates (middle), and information criteria (bottom) of linear mixed effects models predicting visual complexity for Stimulus Set 2.**

| **Parameter** | **Model 0** | **Model 1** | **Model 2** | **Model 3** |
| --- | --- | --- | --- | --- |
| Fixed effects | | | | |
| Intercept | 2.825*** (0.074) | 2.825*** (0.074) | 2.825*** (0.074) | 2.825*** (0.074) |
| MS | –0.327*** (0.031) | –0.327*** (0.055) | –0.327*** (0.031) | –0.327*** (0.057) |
| RMSGIF | 0.546*** (0.031) | 0.546*** (0.031) | 0.546*** (0.044) | 0.546*** (0.046) |
| Random effects | | | | |
| Stimuli |  |  |  |  |
| Intercept | 0.219 | 0.221 | 0.220 | 0.222 |
| Participants |  |  |  |  |
| Intercept | 0.130 | 0.130 | 0.130 | 0.130 |
| MS |  | 0.060*** |  | 0.066*** |
| RMSGIF |  |  | 0.028*** | 0.033*** |
| Residual | 0.607 | 0.546 | 0.578 | 0.513 |
| AIC | 17823 | 17181 | 17559 | 16827 |
| BIC | 17864 | 17236 | 17615 | 16903 |

*Note.* Standard errors are in parentheses. Number of observations = 7308; Number of stimuli = 252; Number of participants = 29. Significance levels of fixed effects are determined using Satterthwaite’s approximation of degrees of freedom. Significance levels of random effects are calculated using likelihood-ratio tests comparing models with corresponding models not including the random effect. (Since linear mixed effects models cannot be calculated without a random intercept, significance levels of random intercepts are not given.)

* *p* < .05, ** *p* < .01, *** *p* < .001.
